# Supplementary material for: Circuit-specific hippocampal ΔFosB underlies resilience to stress-induced social avoidance
Source: Nat Commun. 2020 Sep 8;11:4484. doi: 10.1038/s41467-020-17825-x (PMC7479591; doi:10.1038/s41467-020-17825-x)
Supplement: Supplementary file 1 — Supplementary Information [file 41467_2020_17825_MOESM1_ESM.pdf]

Supplemental Methods

Animals. All experiments were approved by the Institutional Animal Care and Use Committee at Michigan State University in accordance with AAALAC. Male C57Bl/6J mice (3-5/cage; 7-8 wk old upon arrival from Jackson Labs) were allowed at least 5 d to acclimate to the facility prior to any experimental procedures. The *floxed FosB* mouse strain ($FosB_{fl/fl}$)[1] was a generous gift from the laboratory of Dr. Eric Nestler at the Icahn School of Medicine at Mount Sinai, and the $Rosa26_{eGFP\text{-}L10a}$ mice[2] were a generous gift from the laboratory of Dr. Gina Leinninger at Michigan State University. Male CD-1 retired breeder mice (1/cage; age varies upon arrival from Charles River) were allowed at least one week, but more commonly two to three weeks, to acclimate to the facility prior to any experimental procedures. Prior to chronic social defeat stress experiments, CD-1 were allowed 5-7 d to acclimate to the novel housing conditions described below. Unless otherwise stated, all mice were group housed in a 12:12 h light/dark cycle with *ad libitum* food and water. Temperature (22°C) and humidity (50-55%) were held constant in animal housing and behavioral testing rooms.

Surgery & Viral Vectors. Stereotaxic surgery was conducted as previously described[3]. For ΔJunD experiments, viral vectors (AAV2-CMV-GFP or AAV2-CMV-ΔJunD-GFP) were bilaterally infused at two sites in the dHPC (-2.2 AP, ±2.0 ML, -2.1 & -1.9 DV relative to bregma, 10° angle) or vHPC (-3.6 AP, ±3.2 ML, -4.8 & -3.0 DV relative to bregma, 5° angle; 0.3 uL per DV site) to ensure spread of transduction throughout each structure. Experimental procedures commenced at least 4 wks following surgery. For dual virus CRISPR/Cas9 *FosB* gene silencing experiments, Cas9-expressing retrograde vector (HSV-hEf1α-LS1L-myc-Cas9; 0.5 uL) was infused into NAc (+1.6 AP, ±1.5 ML, -4.4 DV relative to bregma, 10° angle) or BLA (-1.6 AP, ±3.4 ML, -4.5 DV relative to bregma, 0° angle). After 3 wks, control viral vector (HSV-IE4/5-TB-eYFP-CMV-IRES-Cre) or *FosB* gRNA (HSV-IE4/5-TB-FosB gRNA-CMV-eYFP-IRES-CRE) were infused into the ventral CA1 region of vHPC (vCA1; -3.4 AP, ±3.2 ML, -4.8 DV relative to

bregma, 3° angle; 0.5 uL). Experimental procedures commenced at least 2 weeks following vHPC surgeries. For rescue experiment, Cre-dependent Cas9 and ΔFosB expressing vector (HSV-hEF1α-LSIL-ΔFosB-myc-Cas9; or Cre-dependent Cas9 alone vector as a control) was infused into NAc (0.5 uL), and following 3 weeks *FosB* gRNA vector was infused into vCA1 (0.5 uL). For ΔFosB overexpression in hippocampal circuits, Cre-dependent ΔFosB vector (HSV-hEF1α-LSIL-ΔFosB-IRES-GFP; 0.5 uL) was infused into NAc or BLA and Cre vector (AAV2-CMV-Cre-GFP; or AAV2-CMV GFP as a control) was infused into vCA1. Experimental procedures commenced at least 4 weeks following surgeries. For electrophysiology of ΔFosB-expressing vHPC neurons, viral vectors (HSV-IE4/5-ΔFosB-CMV-GFP; or HSV-CMV-GFP as control) were bilateral infused into vCA1 (0.5 uL) and experimental procedures commenced 2-4 d following surgery. For electrophysiology of *FosB* KO in vHPC-NAc neurons, Cre expressing retrograde vector (HSV-hEf1α-Cre) was bilaterally infused into NAc (0.5 uL) of *Rosa26*$_{eGFP/L10}$ mice and experimental procedures commenced at least 3 weeks following surgery. AAV viral vectors were obtained from the University of North Carolina at Chapel Hill (UNC Vector Core: https://www.med.unc.edu/genetherapy/vectorcore/) and HSV viral vectors came from Massachusetts General Hospital (Dr. Rachael Neve, Gene Delivery Technology Core: https://researchcores.partners.org/mvvc/about).

Chronic social defeat stress (CSDS). CSDS was performed as previously described[4, 5, 6, 7]. In brief, mice were placed into the home cage of an aggressive retired breeder CD1 mouse containing a perforated plexiglass divider placed between the walls of the cage. The experimental mice were allowed to physically interact with the CD1 for 10 min. Following the aggressive encounter, the mice were placed into the other side of the divider from the CD1 aggressor mouse allowing sensory, but not physical, contact for 24 hours. This protocol was repeated daily for 10 d with a new aggressor every day. Behavioral testing began the day following the final day of stress.

Subchronic defeat stress. Also called a microdefeat, subchronic defeat is an abbreviated social defeat stress protocol[4, 5]. Mice were placed in the home cage of an aggressive retired breeder CD-1 mouse and allowed to interact for 3-5 min, then removed and allowed to rest in their homecage for 15 min. This was repeated for a total of 3 consecutive encounters in a single day. Behavioral testing began the following day.

Behavioral Testing. Behavior was collected using a IR-CCD camera (Panasonic) and analyzed using automated videotracking software (CleverSys). Animals were transported to the behavioral testing rooms in their home cages and allowed 30 min to habituate to the room.

Social Interaction (SI). SI testing was conducted as previously described[4, 6]. Briefly, under red light conditions, mice were placed into the center of a custom made square, opaque arena (38 cm W x 38 cm L x 35 cm H) containing an empty wire mesh cage (10 cm diameter) against one wall and allowed to explore for 150 s. The experimental mice were then removed from the arena and a novel CD1 mouse was placed in the wire mesh cage. Experimental mice were then reintroduced to the arena and allowed to explore for another 150 s. The time spent in proximity (7.5 cm) of the wire mesh object was defined as "interaction zone" time while the time spent in two corners (9 x 9 cm square) farthest from the object were defined as corner zone time. Social interaction (SI) ratio was determined by calculating the time spent in the interaction zone when the CD1 was present divided by the time spent in the interaction zone when the CD1 was absent.

Elevated Plus Maze (EPM). Testing in EPM was conducted as previously described[3]. Briefly, under red light conditions, mice were placed onto the center of an elevated plus maze, with two open arms and two closed arms, and allowed to explore for 5 min. The time spent on the open arms and the number of open arm entries were recorded.

Temporally dissociative passive avoidance (TDPA). TDPA testing was conducted as previously described[3, 8]. Briefly, mice were placed into the lit side of light dark box. After 2 min of exploration, a door allowing entry into the dark side was raised. Upon entry (full body, excluding tail) into the dark side, the door was lowered and, after 5 min, mice received a mild footshock (0.7 mA, 2 s). Mice were returned to their homecage after 30 s. Testing (all of the same conditions including footshock) was repeated daily for 5 d. The latency (s) to "cross over" from the light side to dark side was manually recorded daily.

Open Field (OF). Testing in OF was conducted as previously described[3]. Briefly, under red light conditions, mice were placed into the center of white opaque, square custom-made OF and allowed to explore for 1 h. Time spent in the center zone (50% of the size of OF, centered) and the total distance moved (in cm) were recorded.

von Frey Test for tactile allodynia

Tactile allodynia was assessed using calibrated von Frey filaments as previously described[9]. Mice were habituated to clear plexiglass containers over a mesh floor for 1 h. The next day mice were placed back into the containers for 30 min prior to testing. A series of von Frey filaments were applied to the plantar surface of the hindpaw with sufficient force to bend the filaments for at least 6 s. A paw withdrawal or rapid flinch response was recorded. In the absence of this response, a filament of the next greater force was applied. If a response occurred, the next lower filament was applied. Paw withdrawal threshold (g) was recorded as the force that produced a 50% likelihood of withdrawal.

Novelty-suppressed Feeding

Mice were restricted from ad libitum chow for 24 h prior to testing. Mice were placed into the corner of an OF arena (see above) with one specific difference: a single chow pellet was placed into the center under light conditions. All mice were naïve to the arena. Latency to feed (s) was visually recorded as a measure of novelty-induced suppression of feeding.

Single-trial Contextual Avoidance

Contextual avoidance after a single conditioning trial was assessed under the same condtions as TDPA with one exception: mice were shocked immediately (1 s) after crossover into the dark side of the box. Crossover latency was manuall recorded 24 h later.

Tail Suspension Test

Mice were hung using standard laboratory tape from their tails. Tape was adhered to a horizontal bar. Mice were allowed to hang for 10 minutes. Any mouse that crawled back up its tail was removed from the analysis. Immobility, defined as lack of skeletal movement for at least 1 s, was recorded for the duration of the 10 minutes via automated tracking software (FreezeScan, CleverSys, Inc.)

Immunofluorescent staining for FosB-immunoreactivity. *Rosa26*$_{eGFP/L10a}$ mice underwent stereotaxic surgery to infuse retrograde HSV-hEf1α-Cre into the NAc (see above). After waiting three weeks for full expression, mice were transcardially perfused with cold PBS, followed by 10% formalin. In other experiments, mice received ΔFosB overexpression (see above) and were sacrificed and perfused 4-8 weeks following surgery. Brains from all immunostaining experiments were postfixed 24 h in 10% formalin, cryopreserved in 30% sucrose, and sliced frozen on an SM2010R microtome (Leica) into 35 µm sections. Immunohistochemistry was performed using primary antibodies against FosB (ab11959; 1:1000; Abcam), GFP (ab5450; 1:1000; Abcam), and α2AAR (PA1-048; 1:1000; Invitrogen), and secondary antibodies (1:200; Jackson Immunoresearch) conjugated to fluorescent markers (AlexaFluor 488; Cy3; Cy5). Fluorescent images were visualized on an Olympus FluoView 1000 filter-based laser scanning confocal microscope. Intensity of α2AAR signal in individual cells was quantified using ImageJ software by an experimenter blinded to conditions.

Translational ribosomal affinity purification (TRAP) and cDNA library preparation. Three weeks following injection of retrograde HSV-Cre into NAc, Cre-dependent L10-GFP-expressing mice (*Rosa26*$_{eGFP/L10a}$) were sacrificed and brains were immediately dissected into 1 mm coronal sections. Transduced tissue from ventral hippocampi (vHPC) of both wildtype and *FosB*$_{fl/fl}$ mice was collected using 14 gauge biopsy punches guided by a fluorescent dissecting microscope (Leica) and stored at -80° C until processing (*n* = 3/group, 3-4 mice pooled per *n*). Polyribosome-associated RNA was affinity purified as previously described [10, 11]. Briefly, tissue was homogenized in ice-cold tissue-lysis buffer (20 mM HEPES [pH 7.4], 150 mM KCl, 10 mM MgCl2, 0.5 mM dithiothreitol, 100 µg/ml cycloheximide, protease inhibitors, and recombinant RNase inhibitors) using a motor-driven Teflon glass homogenizer. Homogenates were centrifuged for 10 min at 2000 *g* (4° C), supernatant was supplemented with 1% NP-40 (AG Scientific, #P1505) and 30 mM DHPC (Avanti Polar Lipids, #850306P), and centrifuged again for 10 min at 20000 *g* (4°C). Supernatant was collected and incubated with Streptavidin MyOne T1 Dynabeads (Invitrogen, #65601) that were coated with anti-GFP antibodies (Memorial Sloan-Kettering Monoclonal Antibody Facility; clone names: Htz-GFP-19F7 and Htz-GFP-19C8, 50 ug per antibody per sample) using recombinant biotinylated Protein L (Thermo Fisher Scientific, # 29997) for 16-18 h on a rotator (4° C) in low salt buffer (20 mM HEPES [pH 7.4], 350 mM KCl, 1% NP-40, 0.5 mM dithiothreitol, 100 µg/ml cycloheximide). Beads were isolated and washed with high salt buffer (20 mM HEPES [pH 7.4], 350 mM KCl, 1% NP-40, 0.5 mM dithiothreitol, 100 µg/ml cycloheximide) and RNA was purified using the RNeasy Micro Kit (Qiagen, #74004). In order to increase yield, each RNA sample was initially passed through the Qiagen MinElute™ column 3 times. Following purification, RNA was quantified using a Qubit fluorometer (Invitrogen) and RNA quality was analyzed using a 4200 Agilent Tapestation (Agilent Technologies). cDNA libraries from 5 ng total RNA were prepared using the SMARTer® Stranded Total RNA-Seq Kit (Takara Bio USA, #635005), according to manufacturer's instructions. cDNA libraries were pooled following Qubit measurement and TapeStation analysis, with a final concentration ~7 nM.

Sequencing. Sequencing was performed at the Icahn School of Medicine at Mount Sinai Genomics Core Facility (https://icahn.mssm.edu/research/genomics/core-facility). Raw sequencing reads from mice were mapped to mm9 using TopHat[12] . Counts of reads mapping to genes were obtained using HTSseq-counts software[13] against Gencode vM1 (mm9) annotation. Differential expression was done using the DESeq2 package[14].

Electrophysiology. Whole-cell, *ex vivo* slice electrophysiology was conducted as previously described[15]. All solutions were bubbled with 95% O_2-5% CO_2 throughout the procedure. Mice were anesthetized with isoflurane anesthesia and transcardially perfused with sucrose artificial cerebrospinal fluid (aCSF; in mM: 234 sucrose, 2.5 KCl, 1.25 NaH_2PO_4, 10 $MgSO_4$, 0.5 CaCl, 26 $NaHCO_3$, 11 glucose). Brains were rapidly removed, blocked, and placed in cold sucrose aCSF. Coronal sections (250 μM) containing vHPC were cut on a vibratome (Leica) and transferred to an incubation chamber containing aCSF (in mM: 126 NaCl, 2.5 KCl, 1.25 NaH_2PO_4, 2 MgCl, 2 CaCl, 26 $NaHCO_3$, 10 glucose) held at 34º C for 30 min before moving to aCSF at room temperature until used for recordings. Recordings were made from a submersion chamber perfused with aCSF (2 mL/min) held at 32º C. Borosilicate glass electrodes (3-6 MΩ) were filled with K-gluconate internal solution (in mM: 115 potassium gluconate, 20 KCl, 1.5 MgCl, 10 phosphocreatine-Tris, 2 MgATP, 0.5 Na_3GTP; pH 7.2-7.4; 280-285 mOsm). GFP positive cells in the ventral CA1 region of HPC were visualized using an upright microscope (Olympus) using infrared and epifluorescent illumination. Whole-cell patch-clamp recordings were made from transfected cells using a Multiclamp 700B amplifier and Digidata 1440A digitizer (Molecular Devices) and whole-cell junction potential was not corrected. Traces were sampled (10 kHz), filtered (10 kHz), and digitally stored. Cells with membrane potential more positive than -50 mV or series resistance >20 MΩ were omitted from analysis. Rheobase was measured by giving brief (250 ms) depolarizing (5 pA) steps with 250 ms between steps. Elicited spike number was measured by issuing increasing depolarizing steps (25-300 pA, 500 ms) with 500 ms step intervals. For synaptic recordings of spontaneous excitatory postsynaptic

currents (sEPSCs), cells were held at -80 mV for 2 min. All electrophysiology recordings were made at approximately 30-32 °C by warming the aCSF line with a single inline heater (Warner Instruments).

Immunohistochemistry for detection of ΔFosB following chronic stress or fluoxetine treatment. Immunohistochemistry was performed as previously described[3], and CSDS is described above. A separate cohort of mice received once-daily intraperitoneal injections of fluoxetine (20 mg/kg; dissolved in saline) or saline. For both experiments, mice were sacrificed 24 h following the last injection or defeat episode. Animals were transcardially perfused with cold PBS, followed by 10% formalin. Brains were postfixed 24 h in 10% formalin, cryopreserved in 30% sucrose, followed by slicing on a microtome into 35 μm sections. Immunohistochemistry was performed using anti-FosB primary antibody (2251; 1:500; Cell Signaling) and biotin-conjugated secondary anti-rabbit (BA-1000; 1:1000; Vector) then visualized by 3,3′-diaminobenzidine staining (Vector Laboratories).

Detection of ventral hippocampal afferents to NAc. mCherry expressing retrograde vector (HSV-hEf1α-mCherry) was infused into NAc and perfused coronal sections were taken at 3 wks following surgery according to previously described protocol (see above).

CRISPR Guide RNA design and testing. gRNAs targeting exon 2 of the *FosB* gene were designed using e-CRISP software (www.e-CRISP.org). The top four sequences were:

gRNA1: TACACCGGGAGCCGGAGTCG

gRNA2: TTACGATCTAAAACTTACCT (this gRNA was most effective and was selected for all *in vivo* work described in the current manuscript; also referred to as AJR4 as it was the fourth gRNA produced for our lab)

gRNA3: TCAACATCCGCTAAGGAAGA

gRNA4: CCGTCTTCCTTAGCGGATGT

Each gRNA was tested by transfection in a mammalian expression plasmid also containing Cas9. Briefly, Neuro2a cells (N2a, American Type Culture Collection) were cultured in EMEM (ATCC) supplemented with 10% heat-inactivated fetal bovine serum (ATCC) in a 5% CO_2 humidified atmosphere at 37° C. Cells were plated into 12-well plates, and 24 h later (when cells were ~30% confluent) cells were transiently transfected using Effectene (Qiagen) with a total of 200 ng DNA per well. Cells were transfected with empty vector, Cas9 alone, or Cas9 with a gRNA to be tested. Cells were then serum starved for 24 h, then refed for 4 h to induce *FosB* gene expression. Cells were pelleted, samples were run on gradient polyacrylamide gels and transferred to PVDF membranes, and Western blot was performed using rabbit anti-FosB antibody (2251; 1:500; Cell Signalling) and HRP conjugated anti-rabbit secondary (PI-1000; 1:40,000; Vector). Signal was detected on film and quantified using ImageJ software.

T7 endonculease surveyor analsysis.

T7 surveyor analysis was performed essentially as described[16, 17]. Briefly, Neuro2a cells were plated and transfected as described above, and DNA was extracted using QuickExtract solution (Epicentre Biotechnologies). A region of the *FosB* gene containing the site targeted by our gRNA was amplified by PCR using the following primers:

RB936	GCTTTTCCCGGAGACTACGA
RB937	AAACCAAAGTGCAAACCGAAC

The surveyor nuclease was then used to selectively digest mismatched duplex formed from the PCR products, allowing detection of Cas9 mutated DNA.

Single virus CRISPR/Cas9 *FosB* KO in brain. Male C57Bl/6J mice (8-10 weeks) received surgeries in dHPC (see methods for stereotaxic surgery above) infusing HSV expressing both Cas9 and FosB gRNA (HSV-syn-Cas9-gRNA-IRES-GFP; or HSV-CMV-GFP as a control). Mice were then tested 2 d following surgery for novel object recognition as previously described[3]. Briefly, mice were exposed for 30 min to two similar, familiar objects in an open field over 2 consecutive days. On the next day, a 5 min test for recognition was conducted, where a novel object was placed instead of one of the familiar objects. Time spent in a zone (interaction time) around the novel and familiar object was measured. Following testing, animals were sacrificed by cardiac perfusion and immunofluorescent detection of FosB and GFP expression was conducted (as above).

Validation of dual virus CRISPR/Cas9 *FosB* KO in vHPC projections. Male C57Bl/6J mice (8-10 weeks) received surgeries in NAc or BLA (see methods for stereotaxic surgery above) infusing HSV expressing Cre-dependent Cas9 (see methods for stereotaxic surgery) and 3 weeks later local HSVs expressing Cre and FosB gRNA into vHPC. Four days post gRNA viral injections, mice were sacrificed according to methods for immunohistochemistry above. Coronal sections containing vHPC were stained for Cas9 (in red; 1:500 Diagenode C15200229) and FosB (in cyan; 1:500 ab11959). FosB intensity levels for each Cas9+ neuron were measured using ImageJ, and the numbers of FosB+ and FosB- Cas9-expressing neurons were quantified.

Quantitative PCR from Neuro2A cells. Neuro2A cells were treated and harvested according to prior method (see CRISPR Guide RNA design and testing), except cells were transfected with empty vector (control), ΔFosB, Cas9 + *FosB* gRNA, or ΔJunD. After cells were harvested, RNA was isolated using TriZol (Invitrogen) homogenization and chloroform layer separation. The clear RNA layer was then processed (RNAeasy MicroKit, Qiagen #74004) and analyzed with NanoDrop. A volume of 10uL of RNA was reverse transcribed to cDNA (High Capactiy cDNA Reverse Transcription Kits Applied BioSystens #4368814). Prior to qPCR, cDNA was diluted to 200 uL. The reaction mixture consisted of 10 uL

PowerSYBR Green PCR Master Mix (Applied Biosystems; #436759), 2uL each of forward and reverse primers and water, and 4 uL cDNA template. Samples were then heated to 95 °C for 10 min (Step 1) followed by 40 cycles of 95 °C for 15 s, 60 °C for 15 s, and 72 °C for 15 s (Step 2), and 95 °C for 15 s, 60 °C for 15 s, 65°C for 5s and 95 °C for 5s(Step 3). Analysis was carried out using the ΔΔC(t) method[18]. Samples were normalized to *Gapdh*.

Adra2a
Forward: CAAGATCAACGACCAGAAGT
Reverse: GTCAAGGCTGATGGCGCACAG

Arhgap36
Forward: ACTTAGAGCAGTCCTTGCGG
Reverse: GGTAGAGCTCTGTCCGGCT

Elavl2
Forward: GGTACCGCCGCCAGGAAACACAACTGTCTAATGGG
Reverse: GCGGCCGCACTGAGGACAAGAGCTCATTAGGCTTTGT

Gapdh
Forward: AGGTCGGTGTGAACGGATTTG
Reverse: TGTAGACAATGTAGTTGAGGTCA

Igfbp6
Forward:GGTCTACAGCCCTAAGTGCG
Reverse: AGGGGCCCATCTCACTATCT

Kctd9
Forward: CGGGTCACGCTGTTCTTGA
Reverse: ACAGCACATCATCATCCCTGA

Nefm
Forward: CAGCTACCAGGACACCATCCAG
Reverse: GTGTACAGAGGCCCGGTGAT

Peg10A
Forward: CCGATACACGCGTTTCCAAC
Reverse: TAAAACCCGCCTGTTCCACA

Peg10B
Forward: AATCCTCGTGTGGAACAGGC
Reverse: TCATCATCTTCGGCGTCAGG

Prkcb
Forward: CAGAGATTGCCATCGGTCTGT
Reverse: CCCCTCAGAATCCAGCATCA

Scg5
Forward: ATCAAGGCTACCCAGACCCT
Reverse: GGATTGACACTCCTCCGCTT

Statistics and Reproducibility. For all experiments, alpha criterion was set to 0.05. Social interaction testing was analyzed for SI ratio and interaction zone time. SI ratio was analyzed by independent samples t-tests between groups. Interaction time was analyzed by mixed two-way ANOVAs with Target as the within factor. Omnibus ANOVAs were followed by Holm-Sidak corrected post hoc comparisons between groups. Avoidance learning in the TDPA was analyzed using mixed two-way ANOVAs with Days as the within factor followed by Holm-Sidak corrected post hoc comparisons between groups. EPM and OF behavior was analyzed by independent samples t-tests between groups. For novel object recognition experiment, object interaction time was analyzed using mixed two-way ANOVAs with Object as the within factor followed by Holm-Sidak corrected post hoc comparisons within groups. For all Western blotting and immunohistochemistry results, data were analyzed by independent samples t-tests between groups. Spike number in electrophysiology experiments was analyzed by mixed two-way ANOVAs with Current as the within factor followed by Holm-Sidak corrected post hoc comparisons between groups. I-V curves in electrophysiology experiments were analyzed by mixed two-way ANOVAs with Voltage as the within factor followed by Holm-Sidak corrected post hoc comparisons between groups. For all other electrophysiological measures: rheobase, spike amplitude, spike half-width, sEPSC amplitude, sEPSC frequency, and other cellular properties (Table S1) data were analyzed by independent samples t-tests between groups. Refer to Table S4 and S5 for all omnibus statistical results.

In most cases, behavioral experiments were conducted in no less than two cohorts to ensure reproducibility. For all viral manipulation experiments, confirmation of viral targeting was conducted using antibodies to enhance native GFP signal (e.g. Fig. 1c). Data from mice lacking targeting in a brain region were removed from analyses. All representative images and electrophysiological recording traces were selected based on data representing the mean for each group. Representative micrograph displayed in

Fig. 4e were reproduced in all samples (n=6/group). The same is true in Fig. 5e. FosB staining micrographs displayed in Fig. S1 were replicated; and micrographs and data shown are from a second replication. Representative micrographs in Figs. S4d, S5b, and S8a were replicated in preliminary studies (two replications for S4d; four replications for S5b; one replication for S8a) and reproduced in the final data shown. Representative micrograph shown in Fig. S11b has been replicated multiple times in our lab and published[19]. In addition, all experiments using the methods proposed in Fig. S11a replicated the same pattern of expression shown in S11b.

Data Availability. Sequencing datasets generated during and analyzed during the current study are available in the NIH GEO repository (https://www.ncbi.nlm.nih.gov/geo/) with the accession code GSE137283. All the other data supporting the findings of this study are available within the article and its supplementary information files and from the corresponding author upon reasonable request. A reporting summary for this article is available as a Supplementary Information file.

Supplemental Bibliography

1. Ohnishi YN*, et al.* Generation and validation of a floxed FosB mouse line. *bioRxiv*, (2017).

2. Brown JA*, et al.* Loss of Action via Neurotensin-Leptin Receptor Neurons Disrupts Leptin and Ghrelin-Mediated Control of Energy Balance. *Endocrinology*, (2017).

3. Eagle AL*, et al.* Experience-dependent induction of hippocampal ΔFosB controls learning. *J Neurosci* **35**, 13773-13783 (2015).

4. Krishnan V*, et al.* Molecular adaptations underlying susceptibility and resistance to social defeat in brain reward regions. *Cell* **131**, 391-404 (2007).

5. Golden SA, Covington HE, Berton O, Russo SJ. A standardized protocol for repeated social defeat stress in mice. *Nat Protoc* **6**, 1183-1191 (2011).

6. Berton O*, et al.* Essential role of BDNF in the mesolimbic dopamine pathway in social defeat stress. *Science* **311**, 864-868 (2006).

7. Vialou V*, et al.* Differential induction of FosB isoforms throughout the brain by fluoxetine and chronic stress. *Neuropharmacology* **99**, 28-37 (2015).

8. Eagle AL, Wang H, Robison AJ. Sensitive assessment of hippocampal learning using temporally dissociated passive avoidance task. *Bio Protoc* **6**, (2016).

9. Laumet G*, et al.* G9a is essential for epigenetic silencing of K+ channel genes in acute-to-chronic pain transition. *Nat Neurosci* **18**, 1746-1755 (2015).

10. Heiman M, Kulicke R, Fenster RJ, Greengard P, Heintz N. Cell type-specific mRNA purification by translating ribosome affinity purification (TRAP). *Nat Protoc* **9**, 1282-1291 (2014).

11. Heiman M*, et al.* A translational profiling approach for the molecular characterization of CNS cell types. *Cell* **135**, 738-748 (2008).

12. Trapnell C*, et al.* Differential gene and transcript expression analysis of RNA-seq experiments with TopHat and Cufflinks. *Nat Protoc* **7**, 562 (2012).

13. Anders S, Pyl PT, Huber W. HTSeq—a Python framework to work with high-throughput sequencing data. *Bioinformatics* **31**, 166-169 (2014).

14. Love MI, Huber W, Anders S. Moderated estimation of fold change and dispersion for RNA-seq data with DESeq2. *Genome Biol* **15**, 550 (2014).

15. Eagle AL, Williams ES, Beatty JA, Cox CL, Robison AJ. ΔFosB decreases excitability of dorsal hippocampal CA1 neurons. *eNeuro* **5**, ENEURO.0104-0118.2018 (2018).

16. Vouillot L, Thelie A, Pollet N. Comparison of T7E1 and surveyor mismatch cleavage assays to detect mutations triggered by engineered nucleases. *G3 (Bethesda)* **5**, 407-415 (2015).

17. Guschin DY, Waite AJ, Katibah GE, Miller JC, Holmes MC, Rebar EJ. A Rapid and General Assay for Monitoring Endogenous Gene Modification. In: *Engineered Zinc Finger Proteins: Methods and Protocols* (ed^(eds Mackay JP, Segal DJ). Humana Press (2010).

18. Tsankova NM, Berton O, Renthal W, Kumar A, Neve RL, Nestler EJ. Sustained hippocampal chromatin regulation in a mouse model of depression and antidepressant action. *Nat Neurosci* **9**, 519-525 (2006).

19. Williams ES*, et al.* Androgen-dependent excitability of mouse ventral hippocampal afferents to nucleus accumbens underlies sex-specific susceptibility to stress. *Biological Psychiatry* **87**, 492-501 (2020).

Supplemental Tables & Figures

Table S1: Cellular properties of ventral hippocampal afferents to NAc.

Table S2: List of Top Target Genes

Table S3: Excel File of TRAP Results

Table S4: Summary of Statistics for Main Figures

Table S5: Summary of Statistics for Supplementary Figures

Fig. S1: Social defeat stress induces ΔFosB in ventral hippocampus.

a, Schematic of CSDS and social interaction test. **b,** Representative photomicrographs of coronal sections (4X) stained for ΔFosB in vHPC from control handled and stressed mice, quantified in **c** #*P*=0.0936 (n=12 control, n=8 stress; independent samples t-test compared to control)**. d,** Representative photomicrographs stained for ΔFosB in vHPC subregions (CA1, CA3, DG) from mice treated with chronic saline or fluoxetine, quantified in **e** ***P*=0.0029 for vDG, ****P*<0.0001 for vCA1, ****P*=0.0002 for vCA3 (n=4 saline, n=4 fluoxetine; independent samples t-tests compared to saline). **f,** Quantitative levels of *FosB* and *ΔFosB* mRNA from circuit-specific TRAP-purified vHPC-NAc neurons exposed to CSDS (or control; n=1 sample/group, pooled n=4-5 mice per sample). Fold change mRNA was normalized to *Rbfox3* (which encodes for NeuN) expression.

Fig. S2: Retrograde mCherry expression in vHPC neurons projecting to NAc.

Schematic and coronal section (4X) showing mCherry expression in vHPC of mice infused with retrograde mCherry in NAc 3 weeks prior to sacrifice. Representative micrograph was reproduced in n=3 mice.

Fig. S3: ΔFosB inhibition in ventral hippocampus does not alter locomotor activity or anxiety-like behavior.

a, ΔJunD expression in vHPC caused no differences in time spent in the open arms or entries into the open arms of elevated plus maze (n=11 GFP, n=14 ΔJunD). **b,** ΔJunD expression in vHPC did not alter time spent in the center of an open field or total distance moved (n=11 GFP, n=14 ΔJunD). All graphs are represented as mean ±SEM.

Fig. S4: Development of CRISPR/Cas9 tool to knockout *FosB*.

a, Representative Western blot showing FosB (55 kDa) and ΔFosB (37 kDa) and total protein stain from Neuro2A cells that were not transfected (NT) or transfected with GFP, or Cas9 plus guide RNAs specific to the FosB gene; quantified in **b.** **P*=0.0273 (n=4 wells/group; independent samples t-test). **c,** T7 endonuclease I surveyor analysis of Neuro2A cells from reveals indels (red line) at *FosB* gene when co-transfected with Cas9 and gRNAs 1-3. **d,** Representative images (100x) from dHPC of mice infused with HSV expressing GFP or GFP plus Cas9 and FosB gRNA2 stained for GFP and ΔFosB. Percentage of FosB-positive cells quantified in **e**. ***P*=0.0099 (n=8 GFP, n=17 FosB gRNA; independent samples t-test compared to GFP alone). **f,** Novel object recognition is impaired in mice with CRISPR/Cas9 *FosB* knockout in dorsal hippocampus. **P*=0.0229 (n=20 GFP, n=18 FosB gRNA; two-way ANOVA with Holm-Sidak post-tests compared to Familiar; Note: 3 data points are outside the axis). All graphs are represented as mean ±SEM.

Fig. S5: Validation of circuit-specific *FosB* knockout.

a, Illustration of dual vector experiment design to knockout *FosB* in vHPC neurons projecting to NAc. Retrograde virus expressing Cre-dependent Cas9 is injected into NAc while local vector expressing FosB gRNA and Cre is infused into vHPC. *FosB* knockout (FosB KO, red & green) occurs in co-transduced NAc-projecting vHPC neurons. **b,** Representative images of Cas9 (red, left), FosB (cyan, middle), and merged image from vHPC of mice that receive control (no gRNA) or *FosB* KO vectors (FosB gRNA). Intensity of FosB staining quantified in **c**. ****P*<0.001 (n=149 control cells; n=119 FosB gRNA cells; independent samples t-test compared to control). **d,** Qualitative analysis of number of Cas9+ cells co-expressing FosB in the nucleus. All graphs are represented as mean ±SEM.

Fig. S6: *FosB* knockout in ventral hippocampal circuits on locomotor activity.

a, Dual-vector CRISPR *FosB* knockout in vHPC-NAc neurons reduces total activity in the open field, but not time spent in the center. **P*=0.0253 (n=7 control, n=8 FosB KO; independent samples t-test compared to Control). **b,** Locomotor activity was unaffected by CRISPR *FosB* knockout in vHPC-BLA neurons (n=6 control, n=8 FosB KO). **c-f** A separate cohort of mice with CRISPR *FosB* KO (or control) in vHPC-NAC was exposed to 10 d of chronic social defeat stress (control-no stress, KO-no stress, control-stress, KO-stress). **c,** Stress significantly decreased paw withdrawal threshold, suggesting that stress increases tactile allodynia, however *FosB* KO did not sensitize this response compared to control stressed mice. **P*<0.0122 (n=8 per group; two-way ANOVA with Holm-Sidak comparisons compared to control-no stress) for main effect comparison between Stress vs No Stress (groups combined by stress condition). **d,** Stress also increased the latency to feed in a novel environment. ***P*=0.0047 (n=12 per group; two-way ANOVA with Holm-Sidak comparisons compared to control-no stress) for main effect comparison between Stress vs No Stress (groups combined by stress condition). **e,** Stress increased contextual avoidance following a single conditioning trial. ****P*=0.0002 (n=12 per group; two-way ANOVA with Holm-Sidak comparisons compared to control-no stress) for main effect comparison between Stress vs No Stress (groups combined by stress condition). **f,** No group differences were observed in immobility in the tail suspension test. *P*>0.05 (n=9-12 per group). All graphs are represented as mean ±SEM.

Fig. S7: Dual labeling of vHPC neurons projecting to NAc and BLA

Representative coronal stained sections of hippocampus (10x) from GFP-L10 mice (n=2; top and bottom panels) receiving retrograde HSV-mCherry into BLA (red) and retrograde HSV-Cre into NAc (green). Collaterals to both regions are labeled in yellow and highlighted by white arrows.

Fig. S8: ΔFosB overexpression in vHPC neurons projecting to NAc.

a, Representative coronal vHPC images (20X and 40X) of FosB (red, middle), GFP (green, right), and merged image (left) from vHPC of mice that receive control (GFP; top) or ΔFosB-overexpressing vectors (ΔFosB, bottom). White arrows indicate co-labeled GFP+ ΔFosB-expressing cells. Overexpression of ΔFosB expressed more ΔFosB co-labeled GFP projections compared to control quantified in **b,** ΔFosB significantly increases the % of co-labeling of GFP+ ΔFosB-expressing vHPC-NAc neurons. ****P*<0.0001 (n=5 GFP, n=6 ΔFosB; independent samples t-test compared to GFP). **c,** Quantitative intensity analysis reveals greater ΔFosB signal intensity in GFP+ vHPC-NAc neurons after ΔFosB overexpression. **P*=0.0212 (n=37 GFP cells, n=46 ΔFosB cells; independent samples t-test compared to GFP only). All graphs are represented as mean ±SEM.

Fig. S9: ΔFosB overexpression in ventral hippocampal circuits on locomotor activity.

a, b ΔFosB overexpression in vHPC-NAc does not alter anxiety-like behavior or avoidance learning (n=7 control, n=8 ΔFosB). **c,** Overexpression of ΔFosB in vHPC-NAc reduces total activity in the open field, but not time spent in the center. **P*=0.0333 (n=7 control, n=8 ΔFosB; independent samples t-test compared to GFP). **d,** ΔFosB overexpression in vHPC-BLA does not affect locomotor activity (n=7 mice/group). All graphs are represented as mean ±SEM.

Fig. S10: Membrane, action potential, and synapse properties of ΔFosB overexpressing vCA1 neurons.

a, ΔFosB reduced outward rectification of current-voltage plot. **P*=0.0014 at 10 mV, **P*<0.0001 at 20-40 mV (n=17 GFP cells, n=11 ΔFosB cells; two-way mixed ANOVA with Holm-Sidak post-tests compared to GFP) **b,** Representative current traces of I-V curve. **c,** ΔFosB reduces peak amplitude and half-width of evoked action potentials. ****P*<0.0001 for amplitude and ****P*=0.0009 for half-width (n=17 GFP cells, n=11 ΔFosB cells; independent samples t-test compared to GFP). **d,** Representative current traces for spikes. **e,** ΔFosB reduces sEPSC frequency but does not affect sEPSC peak amplitude. ****P*<0.0001 (n=17 GFP cells, n=10 ΔFosB cells; independent samples t-test compared to GFP). **f,** Representative voltage traces displaying sEPSCs. All graphs are represented as mean ±SEM.

Fig. S11: Cre-driven GFP expression in ventral hippocampus neurons projecting to nucleus accumbens.

a, Schematic of retrograde Cre vector infusion in NAc driving GFP expression in vHPC. **b**, Representative combined micrograph (4X serial combination) of Cre-dependent GFP expression in vHPC (white arrows) and VTA neurons projecting to NAc. **c,** Schematic of non-circuit-specific knockdown of *FosB*. Cross of FosB knockdown is present in all circuits that project to NAc, including, but not limited to, prefrontal cortex (PFC), ventral tegmental area (VTA), BLA, and vHPC. **d,** Non-circuit-specific knockdown of *FosB* did not affect stress-induced social avoidance. Retrograde Cre-driven knockdown (KD) of FosB in all NAc-projecting neurons in WT and heterozygous floxed *FosB* ($FosB_{fl/-}$) mice. Non-circuit-specific FosB KD did not enhance stress-induced social avoidance. *P*>0.05 compared to WT (n=7 WT, n=7 KD). All graphs are represented as mean ±SEM.

Fig. S12: Membrane, action potential, and synapse properties of vHPC-NAc neurons following *FosB* knockout.

a, *FosB* KO increased outward rectification of current-voltage plot.. **P*=0.0280 at -10 mV, **P*=0.0004 at 0 mV, **P*<0.0001 at 10-40 mV (n=20 WT cells, n=27 KO cells; two-way mixed ANOVA with Holm-Sidak post-tests compared to WT). **b,** Representative current traces of I-V curve. **c,** *FosB* KO decreased half-width but had no effect on amplitude of evoked action potentials. ****P*=0.0006 (n=20 WT cells, n=27 KO cells; independent samples t-test compared to WT; Note: 1 data point is outside the axis in left graph (amplitude), and 1 data point is outside the axis in right graph (half-width)). **d,** Representative current traces for spikes. **e,** *FosB* KO decreased amplitude and increased frequency of sEPSCs. ***P*=0.0051 for frequency and ****P*=0.0004 for amplitude (n=19 WT cells, n=27 KO cells; independent samples t-test compared to WT). **f,** Representative voltage traces displaying sEPSCs. All graphs are represented as mean ±SEM.

Fig. S13: Validation of TRAP-RNA sequencing in ventral hippocampus

a, Visualization of mRNA reads from input and vHPC-NAc TRAP-Seq of a non neuron-specific (*Actb*) and neuron-specific (*Kalrn*) gene. **b,** Visualization of mRNA reads of *Adra2a* gene from WT ($FosB_{+/+}$) and *floxed FosB* ($FosB_{fl/fl}$) mice.

Table S1: Cellular properties of ventral hippocampus neurons.

Measure:	Groups: GFP	vs ΔFosB	WT	vs *FosB* KO
**V_m**	-64.2 (3.0)	-63.3 (0.9)	-65.8 (0.6)	-68.4 (1.0)
**R_m**	83.1 (48.9)	110.2 (19.6)	88.4 (8.2)	112.0 (39.3)
**R_a**	14.1 (3.1)	16.4 (1.5)	18.2 (1.6)	17.9 (0.7)
**C_m**	*78.7 (26.9)*	*59.7 (5.7)**	*55.9 (3.6)*	*72.7 (3.8)**
**Sag**	*0.934 (.026)*	*0.961 (.005)**	*0.898 (.012)*	*0.934 (.008)**

Abbreviations: V_m = resting membrane potential; R_m = membrane resistance; R_a = access resistance; C_m = membrane capacitance; Sag = sag ratio

All data are represented as Mean (±SEM). *P<0.05 by independent samples t-test

Table S2: List of top 25 target genes from circuit-specific TRAP

Gene	Ensemble Gene ID	WT-TRAP % Error	KO-TRAP % Error	WT Enriched?	KO Enriched?	Reg	Fold Change	Sig. (P Value)
Peg10	ENSMUSG00000092035	3.82	2.00	FALSE	TRUE	Up	1.00	<0.001
Igfbp6	ENSMUSG00000023046	5.90	16.64	TRUE	FALSE	Down	-0.54	<0.001
Scg5	ENSMUSG00000023236	10.95	1.11	FALSE	TRUE	Down	-0.52	<0.001
Siae	ENSMUSG00000001942	41.37	8.78	FALSE	TRUE	Up	0.51	<0.001
Arhgap36	ENSMUSG00000036198	34.61	16.32	FALSE	TRUE	Up	0.49	<0.001
Dusp4	ENSMUSG00000031530	6.32	11.79	TRUE	FALSE	Down	-0.47	0.001
Zcchc12	ENSMUSG00000036699	7.17	3.26	FALSE	TRUE	Up	0.45	<0.001
Fosb	ENSMUSG00000003545	10.33	14.46	TRUE	FALSE	Down	-0.44	0.002
Kcng1	ENSMUSG00000074575	9.46	5.50	FALSE	TRUE	Up	0.44	<0.001
Nefm	ENSMUSG00000022054	7.32	2.81	TRUE	FALSE	Down	-0.44	<0.001
Ier5	ENSMUSG00000056999	11.97	15.58	TRUE	FALSE	Down	-0.43	0.003
Timm50	ENSMUSG00000003438	4.40	1.68	TRUE	TRUE	Down	-0.43	0.002
Gpr101	ENSMUSG00000036357	5.90	28.32	FALSE	TRUE	Up	0.42	0.004
Adra2a	ENSMUSG00000033717	11.01	10.10	FALSE	TRUE	Up	0.40	0.005
Sstr1	ENSMUSG00000035431	5.03	9.45	FALSE	TRUE	Up	0.40	0.006
Grp	ENSMUSG00000024517	16.83	5.80	TRUE	TRUE	Up	0.40	0.005
Peli3	ENSMUSG00000024901	5.87	2.41	FALSE	TRUE	Up	0.40	0.006
Poll	ENSMUSG00000025218	16.57	26.40	FALSE	TRUE	Up	0.40	0.005
Pdcd6	ENSMUSG00000021576	5.34	7.11	TRUE	FALSE	Down	-0.39	0.004
Rwdd2a	ENSMUSG00000032417	9.60	4.95	FALSE	TRUE	Up	0.39	0.004
Scube1	ENSMUSG00000016763	32.20	3.71	TRUE	TRUE	Down	-0.39	0.008
Grasp	ENSMUSG00000000531	10.72	6.05	TRUE	TRUE	Down	-0.39	0.001
Ap2s1	ENSMUSG00000008036	12.51	2.41	TRUE	TRUE	Down	-0.38	0.001
Scamp4	ENSMUSG00000079020	8.51	7.92	TRUE	FALSE	Down	-0.38	0.009
Rab3b	ENSMUSG00000003411	5.97	15.06	FALSE	TRUE	Up	0.38	0.003

Sig. (P Value) was based on independent samples t-test.

Table S4: Summary of Statistics for Main Figures

Figure	Test	Comparison	Stat	p-value	Summary
**Fig 1b**	Independent samples t-test	Control vs Stress	t (254) = 2.847	P = 0.0048	**
**Fig 1e (Left)**	Independent samples t-test	GFP vs ΔJunD	t (35) = 2.632	P = 0.0125	*
**Fig 1e (Right)**	2-way Mixed ANOVA	Group: GFP vs ΔJunD	F (1, 35) = 1.790	P = 0.1896	NS
		Trial: No Target vs Target	F (1, 35) = 21.16	P < 0.0001	***
		Group X Trial	F (1, 35) = 9.955	P = 0.0033	**
**Fig 1f (Left)**	Independent samples t-test	GFP vs ΔJunD	t (16) = 0.4163	P = 0.6827	NS
**Fig 1f (Right)**	2-way Mixed ANOVA	Group: GFP vs ΔJunD	F (1, 16) = 0.7639	P = 0.3950	NS
		Trial: No Target vs Target	F (1, 16) = 2.378	P = 0.1426	NS
		Group X Trial	F (1, 16) = 0.0928	P = 0.7646	NS
**Fig 2b**	Independent samples t-test	Control vs FosB KO	t (13) = 3.056	P = 0.0092	**
**Fig 2c**	2-way Mixed ANOVA	Group: Control vs FosB KO	F (1, 13) = 0.343	P = 0.5682	NS
		Day: (1,2,3,4,5)	F (4, 52) = 12.150	P < 0.0001	***
		Group X Day	F (4, 52) = 0.337	P = 0.8516	NS
**Fig 2d (Left)**	Independent samples t-test	Control vs FosB KO	t (13) = 0.144	P = 0.8877	NS
**Fig 2d (Right)**	Independent samples t-test	Control vs FosB KO	t (13) = 2.082	P = 0.0576	NS
**Fig 2f**	Independent samples t-test	Control vs FosB KO	t (19) = 0.872	P = 0.3939	NS
**Fig 2g**	2-way Mixed ANOVA	Group: Control vs FosB KO	F (1, 12) = 3.896	P = 0.0719	NS
		Day: (1,2,3,4,5)	F (4, 48) = 9.332	P < 0.0001	***
		Group X Day	F (4, 48) = 2.667	P = 0.0434	*
**Fig 2h (Left)**	Independent samples t-test	Control vs FosB KO	t (11) = 3.241	P = 0.0079	**
**Fig 2h (Right)**	Independent samples t-test	Control vs FosB KO	t (11) = 3.956	P = 0.0022	**
**Fig 3b (Left)**	Independent samples t-test	KO vs Rescue	t (23) = 2.597	P = 0.0161	*
**Fig 3b (Right)**	2-way Mixed ANOVA	Group: KO vs Rescue	F (1, 23) = 0.9440	P = 0.3414	NS
		Trial: No Target vs Target	F (1, 23) = 2.495	P = 0.1278	NS
		Group X Trial	F (1, 23) = 6.452	P = 0.0183	*
**Fig 3d (Left)**	Independent samples t-test	KO vs Rescue	t (25) = 3.154	P = 0.0042	**
**Fig 3d (Right)**	Independent samples t-test	KO vs Rescue	t (25) = 1.841	P = 0.0776	NS
**Fig 3e**	2-way Mixed ANOVA	Group: KO vs Rescue	F (1, 26) = 10.45	P = 0.0033	**
		Day (1,2,3,4,5)	F (4, 104) = 20.11	P < 0.0001	***
		Group X Day	F (4, 104) = 4.828	P = 0.0013	**
**Fig 3g (Left)**	Independent samples t-test	Control vs ΔFosB	t (31) = 2.575	P = 0.0150	*
**Fig 3g (Right)**	2-way Mixed ANOVA	Group: Control vs ΔFosB	F (1, 31) = 1.703	P = 0.2015	NS
		Trial: No Target vs Target	F (1, 31) = 2.073	P = 0.1600	NS

		Group X Trial	F (1, 31) = 4.533	P = 0.0413	*
**Fig 3i (Left)**	Independent samples t-test	Control vs ΔFosB	t (12) = 0.310	P = 0.7619	NS
**Fig 3i (Right)**	Independent samples t-test	Control vs ΔFosB	t (12) = 1.244	P = 0.2371	NS
**Fig 3j**	2-way Mixed ANOVA	Group: Control vs ΔFosB	F (1, 12) = 0.5060	P = 0.4905	NS
		Day (1,2,3,4,5)	F (4, 48) = 13.91	P < 0.0001	***
		Group X Day	F (4, 48) = 0.5236	P = 0.7189	NS
**Fig 4b (main)**	2-way Mixed ANOVA	Group: GFP vs ΔFosB	F (1, 26) = 7.540	P = 0.0108	*
		Current	F (11, 286) = 145.6	P < 0.0001	***
		Group X Current	F (11, 286) = 7.590	P < 0.0001	***
**Fig 4b (inset)**	Independent samples t-test	GFP vs ΔFosB	t (26) = 2.476	P = 0.0108	*
**Fig 4c**	Independent samples t-test	GFP vs ΔFosB	t (26) = 1.905	P = 0.0680	NS
**Fig 4f**	Independent samples t-test	WT vs KO	t (10) = 3.603	P = 0.0048	**
**Fig 4h (main)**	2-way Mixed ANOVA	Group: WT vs KO	F (1, 45) = 4.391	P = 0.0418	*
		Current	F (11, 495) = 294.4	P < 0.0001	***
		Group X Current	F (11, 495) = 1.694	P = 0.0715	NS
**Fig 4h (inset)**	Independent samples t-test	WT vs KO	t (45) = 2.095	P = 0.0418	*
**Fig 4i**	Independent samples t-test	WT vs KO	t (45) = 0.7551	P = 0.4541	NS
**Fig 5c (Adra2a)**	Independent samples t-test	Control vs ΔFosB	t (22) = 2.4239	P = 0.0240	*
**Fig 5c (Arhgap36)**	Independent samples t-test	Control vs ΔFosB	t (10) = 0.6722	P = 0.5167	NS
**Fig 5c (Elavl2)**	Independent samples t-test	Control vs ΔFosB	t (10) = 1.0636	P = 0.3125	NS
**Fig 5c (KCTD9)**	Independent samples t-test	Control vs ΔFosB	t (9) = 1.1526	P = 0.2788	NS
**Fig 5c (Nefm)**	Independent samples t-test	Control vs ΔFosB	t (22) = 1.7655	P = 0.0914	NS
**Fig 5c (Gaa)**	Independent samples t-test	Control vs ΔFosB	t (10) = 0.4133	P = 0.6881	NS
**Fig 5c (Igfbp6)**	Independent samples t-test	Control vs ΔFosB	t (10) = 1.7649	P = 0.1081	NS
**Fig 5c (Peg10A)**	Independent samples t-test	Control vs ΔFosB	t (10) = 1.7305	P = 0.1142	NS
**Fig 5c (Peg10B)**	Independent samples t-test	Control vs ΔFosB	t (10) = 0.0383	P = 0.9702	NS
**Fig 5c (Prkcb)**	Independent samples t-test	Control vs ΔFosB	t (10) = 1.9366	P = 0.0815	NS
**Fig 5c (Scg5)**	Independent samples t-test	Control vs ΔFosB	t (10) = 1.1394	P = 0.2811	NS
**Fig 5d**	One-way ANOVA	Group: Control, Cas9, ΔJunD	F (2, 33) = 4.273	P = 0.0224	*
**Fig 5f**	Independent samples t-test	FosB-/- vs FosBfl/fl	t (181) = 6.203	P < 0.0001	***

Table S5: Summary of Statistics for Supplementary Figures

Figure	Test	Comparison	Stat	p-value	Summary
**Fig S1c (Left)**	Independent samples t-test	Control vs Defeat	t (18) = 1.770	P = 0.0936	NS
**Fig S1e (Left)**	Independent samples t-test	Saline vs Fluoxetine	t (10) = 3.908	P = 0.0029	**
**Fig S1e (Middle)**	Independent samples t-test	Saline vs Fluoxetine	t (10) = 7.951	P < 0.0001	***
**Fig S1e (Right)**	Independent samples t-test	Saline vs Fluoxetine	t (10) = 5.674	P = 0.0002	***
**Fig S3a (Left)**	Independent samples t-test	GFP vs ΔJunD	t (23) = 1.517	P = 0.1430	NS
**Fig S3a (Right)**	Independent samples t-test	GFP vs ΔJunD	t (23) = 1.118	P = 0.2750	NS
**Fig S3b (Left)**	Independent samples t-test	GFP vs ΔJunD	t (23) = 1.494	P = 0.1489	NS
**Fig S3b (Right)**	Independent samples t-test	GFP vs ΔJunD	t (23) = 1.240	P = 0.2275	NS
**Fig S4b**	Independent samples t-test	NT vs GFP	t (6) = 0.0616	P = 0.9529	NS
**Fig S4b**	Independent samples t-test	NT vs gRNA1	t (6) = 1.529	P = 0.1771	NS
**Fig S4b**	Independent samples t-test	NT vs gRNA2	t (6) = 2.901	P = 0.0273	*
**Fig S4e**	Independent samples t-test	GFP vs FosB gRNA	t (23) = 2.813	P = 0.0099	**
**Fig S4f**	2-way Mixed ANOVA	Group: GFP vs FosB gRNA	F (1, 36) = 0.5372	P = 0.4684	NS
		Object: Familiar vs Novel	F (1, 36) = 1.109	P = 0.2993	NS
		Group X Object	F (1, 36) = 6.821	P = 0.0131	*
**Fig S5c**	Independent samples t-test	Control vs FosB gRNA	t (266) = 5.876	P < 0.0001	***
**Fig S6a (Left)**	Independent samples t-test	Control vs FosB KO	t (13) = 1.203	P = 0.2504	NS
**Fig S6a (Right)**	Independent samples t-test	Control vs FosB KO	t (13) = 2.527	P = 0.0253	*
**Fig S6b (Left)**	Independent samples t-test	Control vs FosB KO	t (12) = 0.930	P = 0.3706	NS
**Fig S6b (Right)**	Independent samples t-test	Control vs FosB KO	t (12) = 1.341	P = 0.2049	NS
**Fig S6c**	2-way ANOVA	Group: Control vs FosB KO	F (1, 28) = 0.031	P = 0.8607	NS
		Stress: No Stress vs. Stress	F (1, 28) = 7.186	P = 0.0122	*
		Group X Stress	F (1, 28) = 1.918	P = 1.770	NS
**Fig S6d**	2-way ANOVA	Group: Control vs FosB KO	F (1, 44) = 0.120	P = 0.7305	NS
		Stress: No Stress vs. Stress	F (1, 44) = 8.891	P = 0.0047	**
		Group X Stress	F (1, 44) = 0.4044	P = 0.5281	NS
**Fig S6e**	2-way ANOVA	Group: Control vs FosB KO	F (1, 44) = 5.325	P = 0.0258	*
		Stress: No Stress vs. Stress	F (1, 44) = 16.510	P = 0.0002	***
		Group X Stress	F (1, 44) = 1.956	P = 0.1690	NS
**Fig S6f**	2-way ANOVA	Group: Control vs FosB KO	F (1, 38) = 0.155	P = 0.6962	NS
		Stress: No Stress vs. Stress	F (1, 38) = 0.597	P = 0.4447	NS

		Group X Stress	F (1, 38) = 0.103	P = 0.7497	NS
**Fig S8b**	Independent samples t-test	GFP vs ΔFosB	t (9) = 8.407	P < 0.0001	***
**Fig S8c**	Independent samples t-test	GFP vs ΔFosB	t (81) = 2.350	P = 0.0212	*
**Fig S9a (Left)**	Independent samples t-test	Control vs ΔFosB	t (13) = 1.359	P = 0.1972	NS
**Fig S9a (Right)**	Independent samples t-test	Control vs ΔFosB	t (13) = 0.114	P = 0.9113	NS
**Fig Sb**	2-way Mixed ANOVA	Group: Control vs ΔFosB	F (1, 13) = 1.473	P = 0.2465	NS
		Day (1,2,3,4,5)	F (4, 52) = 17.96	P < 0.0001	***
		Group X Day	F (4, 52) = 0.9139	P = 0.4629	NS
**Fig S9c (Left)**	Independent samples t-test	Control vs ΔFosB	t (13) = 0.194	P = 0.8493	NS
**Fig S9c (Right)**	Independent samples t-test	Control vs ΔFosB	t (13) = 2.380	P = 0.0333	*
**Fig S9d (Left)**	Independent samples t-test	Control vs ΔFosB	t (12) = 0.601	P = 0.5590	NS
**Fig S9d (Right)**	Independent samples t-test	Control vs ΔFosB	t (12) = 0.599	P = 0.5603	NS
**Fig S10a**	2-way Mixed ANOVA	Group: GFP vs ΔFosB	F (1, 24) = 12.37	P = 0.0018	**
		Voltage	F (14, 336) = 100.8	P < 0.0001	***
		Group X Voltage	F (14, 336) = 10.27	P < 0.0001	***
**Fig S10b (Left)**	Independent samples t-test	GFP vs ΔFosB	t (26) = 5.479	P < 0.0001	***
**Fig S10b (Right)**	Independent samples t-test	GFP vs ΔFosB	t (26) = 3.760	P = 0.0009	***
**Fig S10c (Left)**	Independent samples t-test	GFP vs ΔFosB	t (25) = 0.3472	P = 0.3472	NS
**Fig S10c (Right)**	Independent samples t-test	GFP vs ΔFosB	t (25) = 5.208	P < 0.0001	***
**Fig S11c**	Independent samples t-test	WT vs Het	t (12) = 1.533	P = 0.1511	NS
**Fig S12a**	2-way Mixed ANOVA	Group: WT vs KO	F (1, 24) = 12.37	P = 0.0018	**
		Voltage	F (14, 336) = 100.8	P < 0.0001	***
		Group X Voltage	F (14, 336) = 10.27	P < 0.0001	***
**Fig S12b (Left)**	Independent samples t-test	WT vs KO	t (45) = 1.553	P = 0.1275	NS
**Fig S12b (Right)**	Independent samples t-test	WT vs KO	t (45) = 3.265	P = 0.0006	***
**Fig S12c (Left)**	Independent samples t-test	WT vs KO	t (44) = 3.823	P = 0.0004	***
**Fig S12c (Right)**	Independent samples t-test	WT vs KO	t (44) = 2.951	P = 0.0051	**

Fig. S1

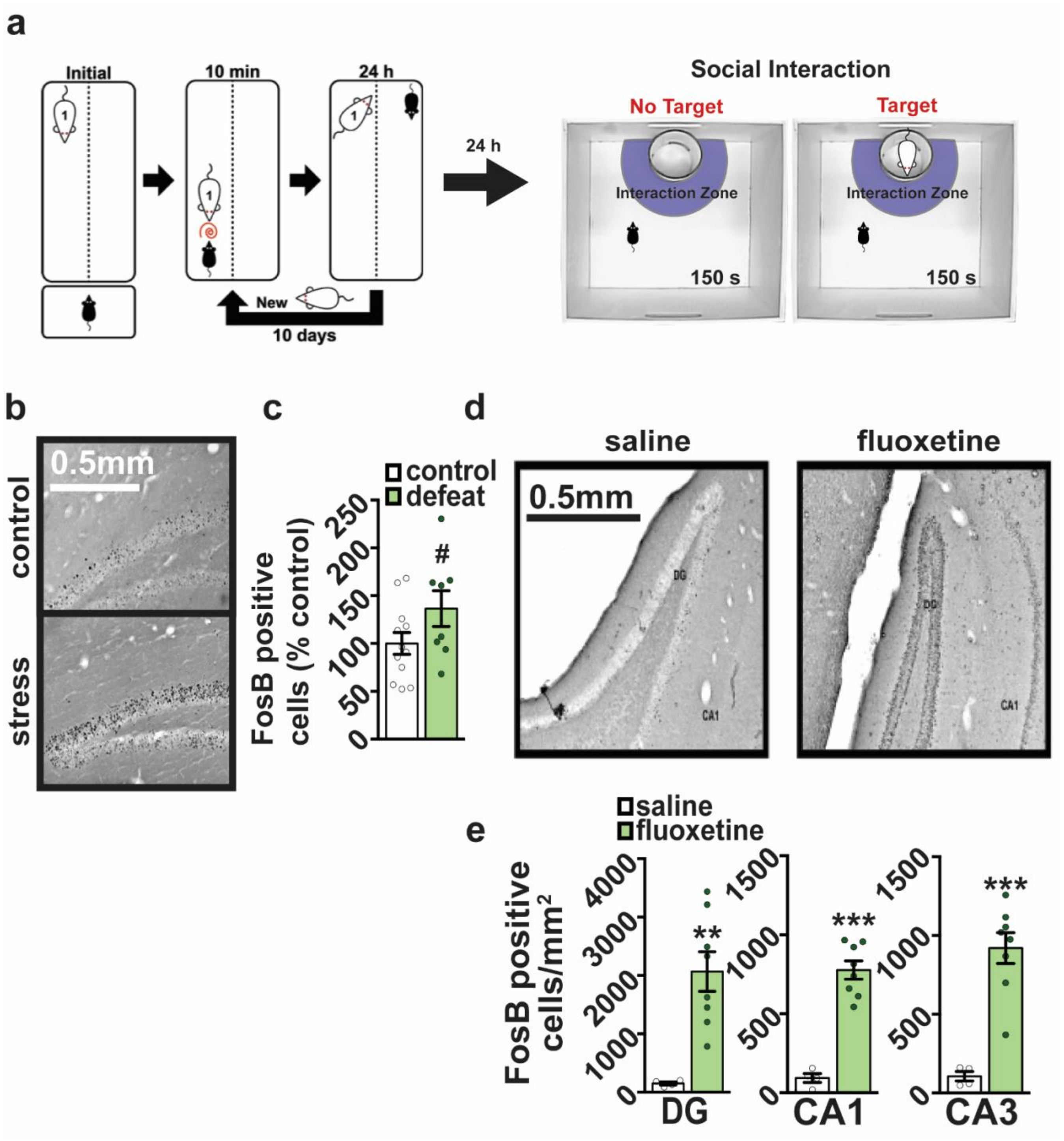

a
Initial
10 min
24 h
1
New
10 days
24 h
Social Interaction
No Target
Target
Interaction Zone
Interaction Zone
150 s
150 s
b
control
stress
0.5mm
c
control
defeat
FosB positive cells (% control)
250
200
150
100
50
0
#
d
saline
fluoxetine
0.5mm
DG
CA1
e
saline
fluoxetine
FosB positive cells/mm2
4000
3000
2000
1000
0
1500
1000
500
**
***
***
DG
CA1
CA3

Fig. S2

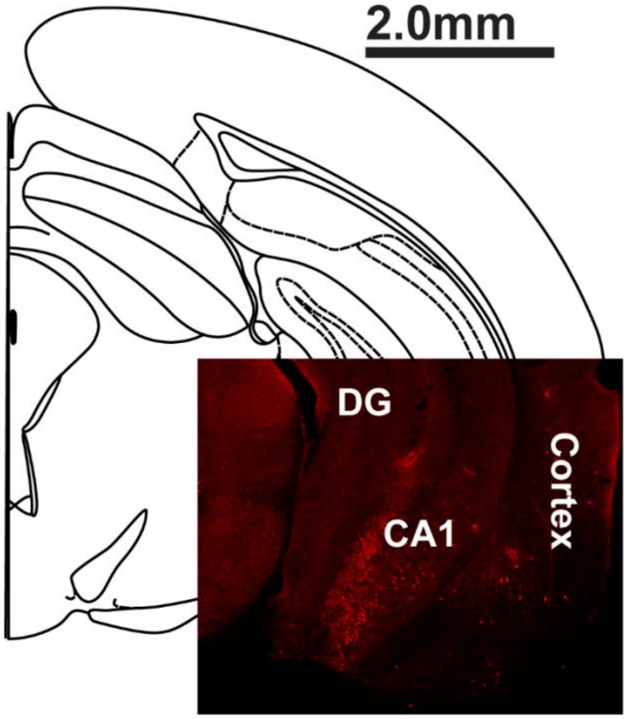

Fig. S3

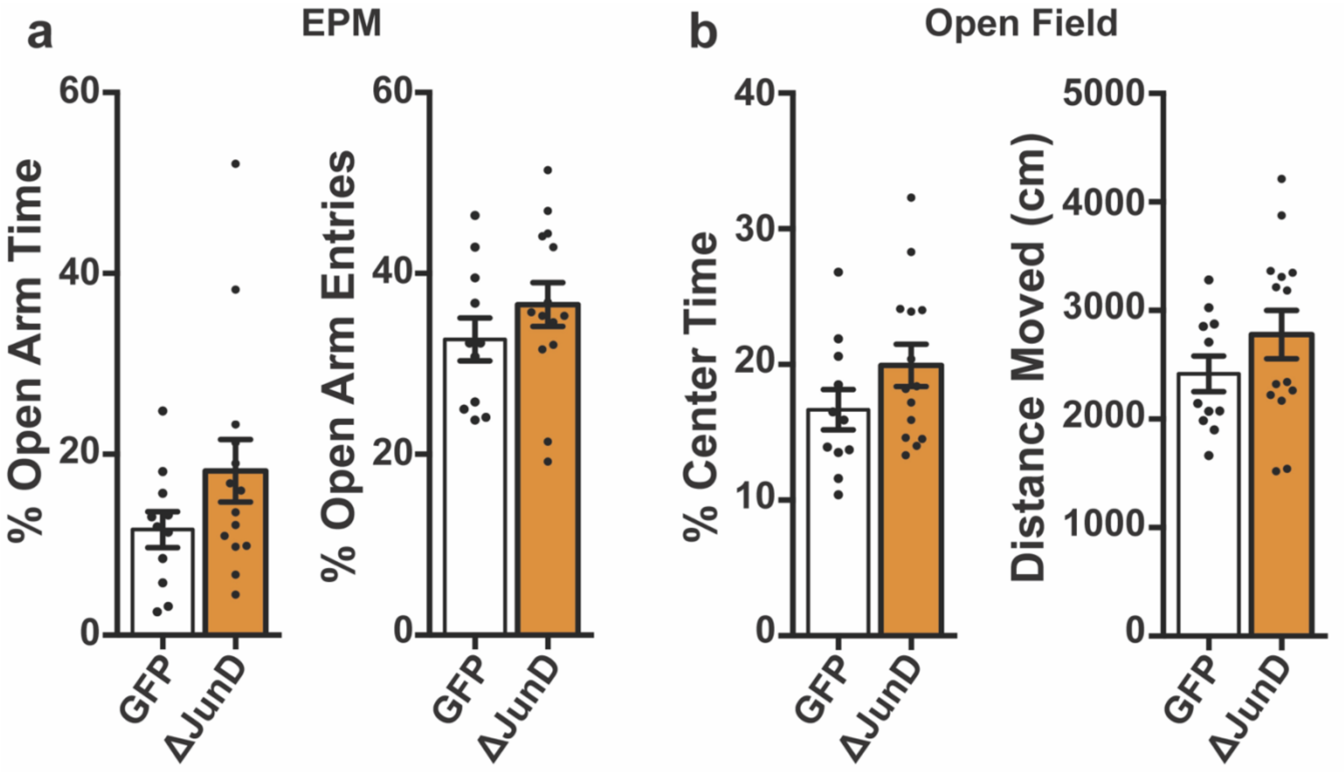

a
EPM
b
Open Field
% Open Arm Time
% Open Arm Entries
% Center Time
Distance Moved (cm)
60
40
20
0
40
30
10
5000
4000
3000
2000
1000
GFP
ΔJunD

Fig. S4

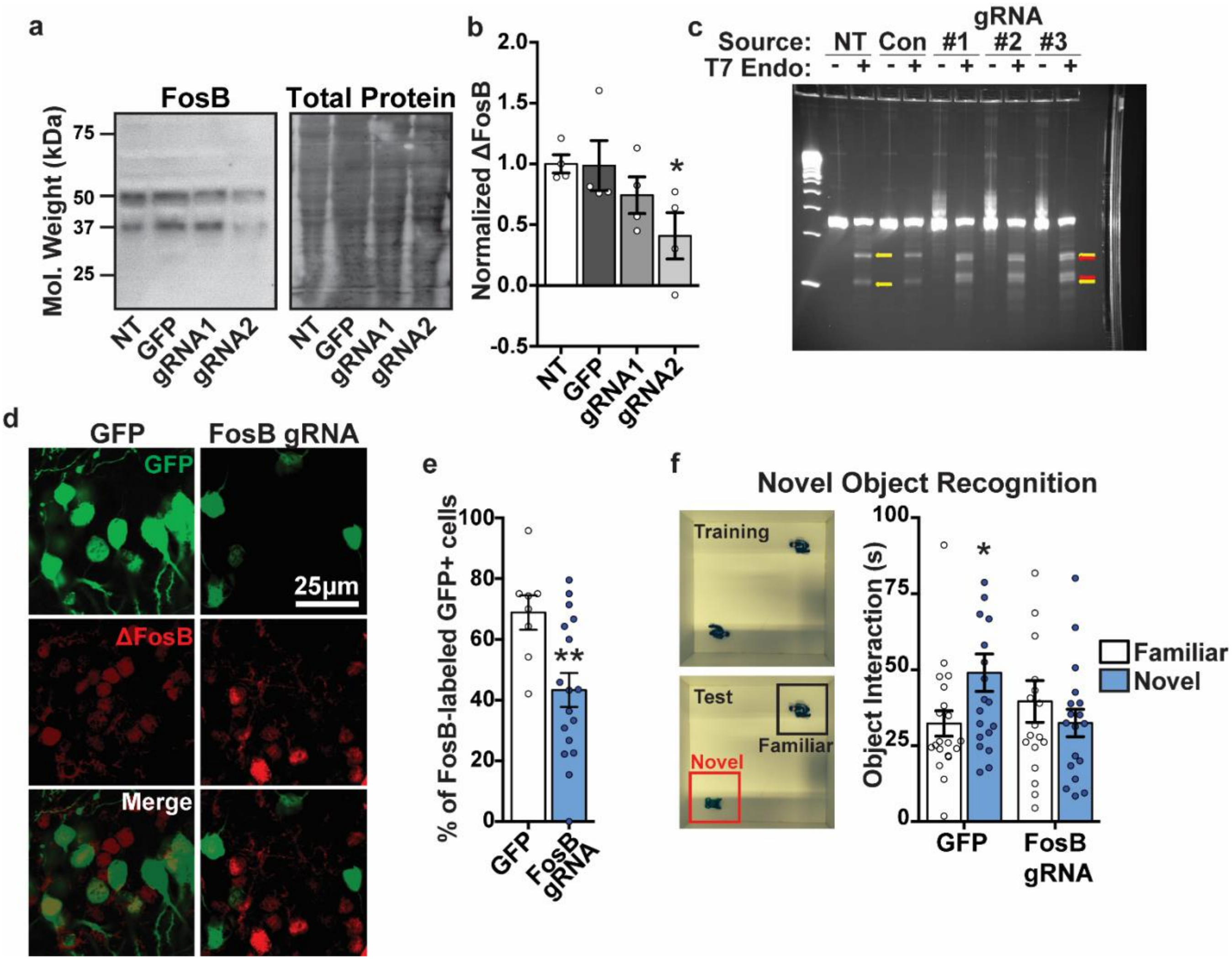
a
FosB
Total Protein
Mol. Weight (kDa)
75
50
37
25
NT
GFP
gRNA1
gRNA2
b
Normalized ΔFosB
2.0
1.5
1.0
0.5
0.0
-0.5
*
c
Source:
NT
Con
gRNA
#1
#2
#3
T7 Endo:
- + - + - + - + - +
d
GFP
FosB gRNA
GFP
25μm
ΔFosB
Merge
e
% of FosB-labeled GFP+ cells
100
80
60
40
20
0
**
GFP
FosB gRNA
f
Novel Object Recognition
Training
Test
Familiar
Novel
Object Interaction (s)
100
75
50
25
0
*
Familiar
Novel
GFP
FosB gRNA

Fig. S5

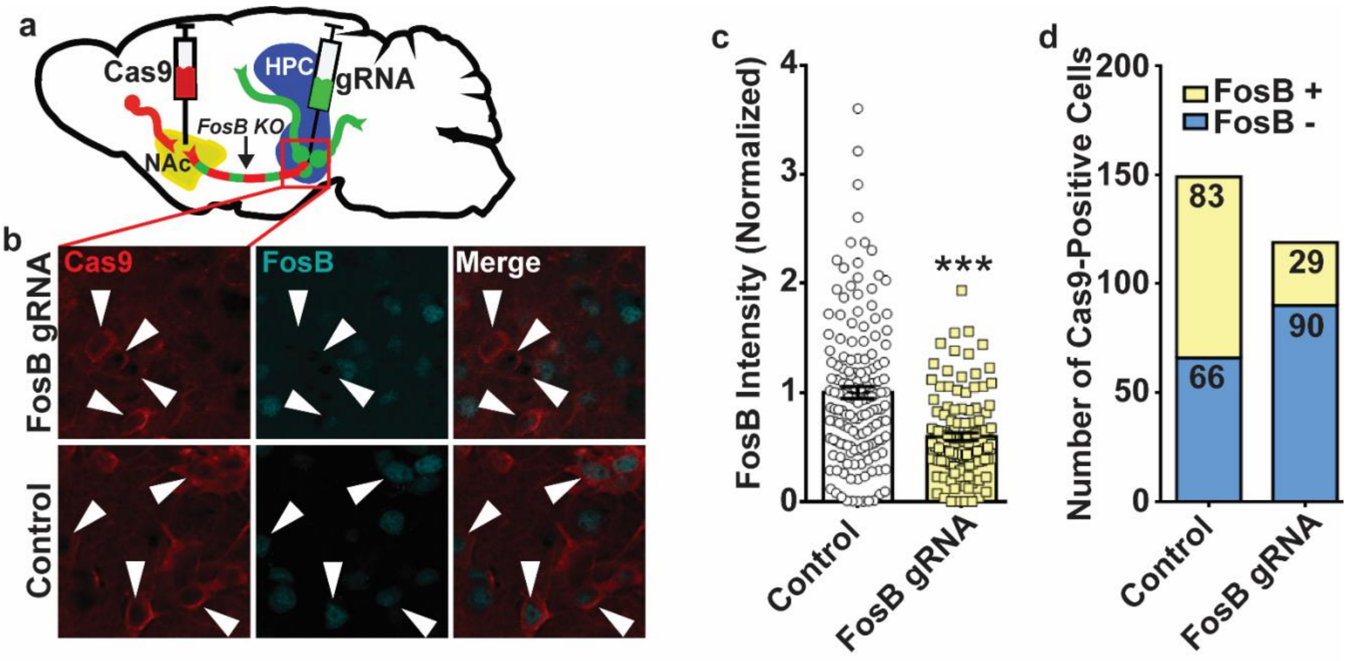
a
Cas9
HPC
gRNA
FosB KO
NAc
b
Cas9
FosB
Merge
FosB gRNA
Control
c
FosB Intensity (Normalized)
4
3
2
1
0
***
Control
FosB gRNA
d
Number of Cas9-Positive Cells
200
150
100
50
0
FosB +
FosB -
83
66
29
90
Control
FosB gRNA

Fig. S6

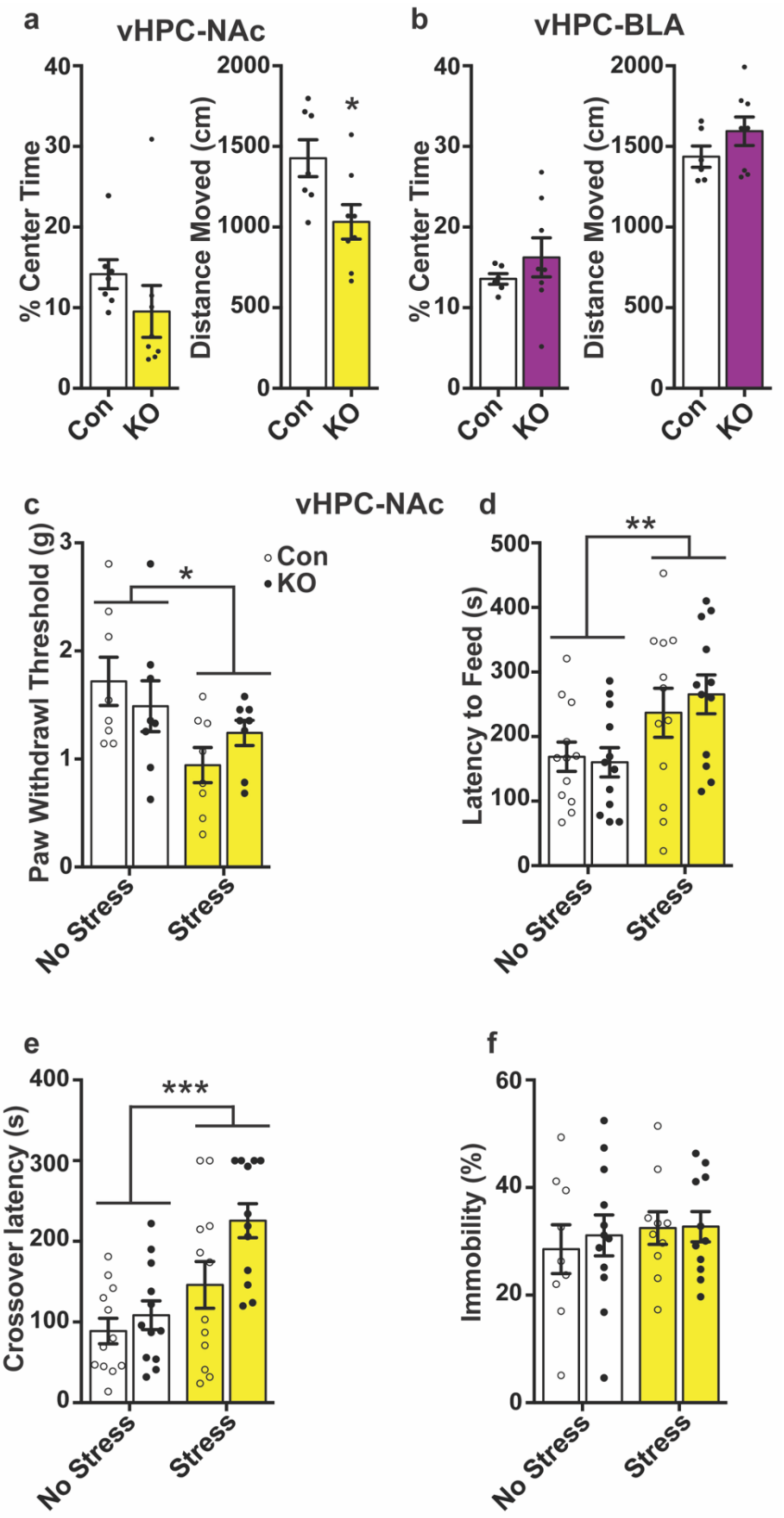
a
vHPC-NAc
% Center Time
Distance Moved (cm)
Con
KO
*
b
vHPC-BLA
c
vHPC-NAc
Paw Withdrawl Threshold (g)
Con
KO
No Stress
Stress
d
Latency to Feed (s)
**
e
Crossover latency (s)
***
f
Immobility (%)

Fig. S7

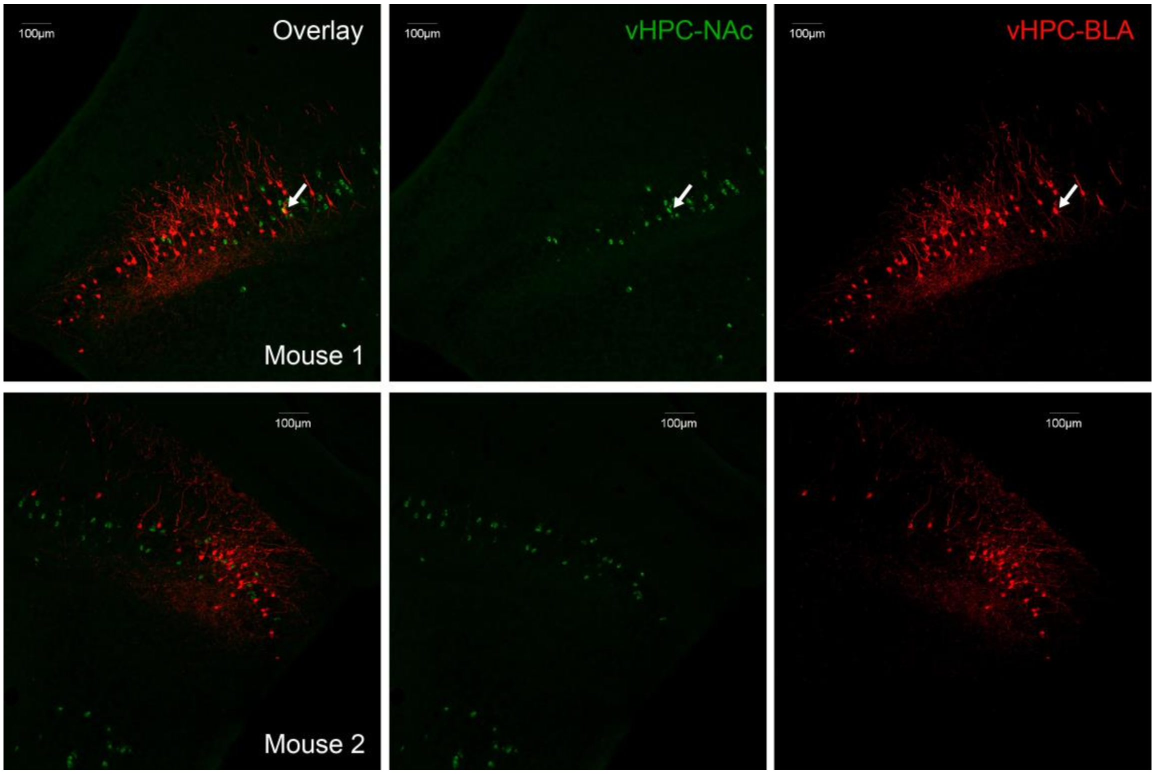
100µm
Overlay
Mouse 1
100µm
vHPC-NAc
100µm
vHPC-BLA
100µm
Mouse 2
100µm
100µm

Fig. S8

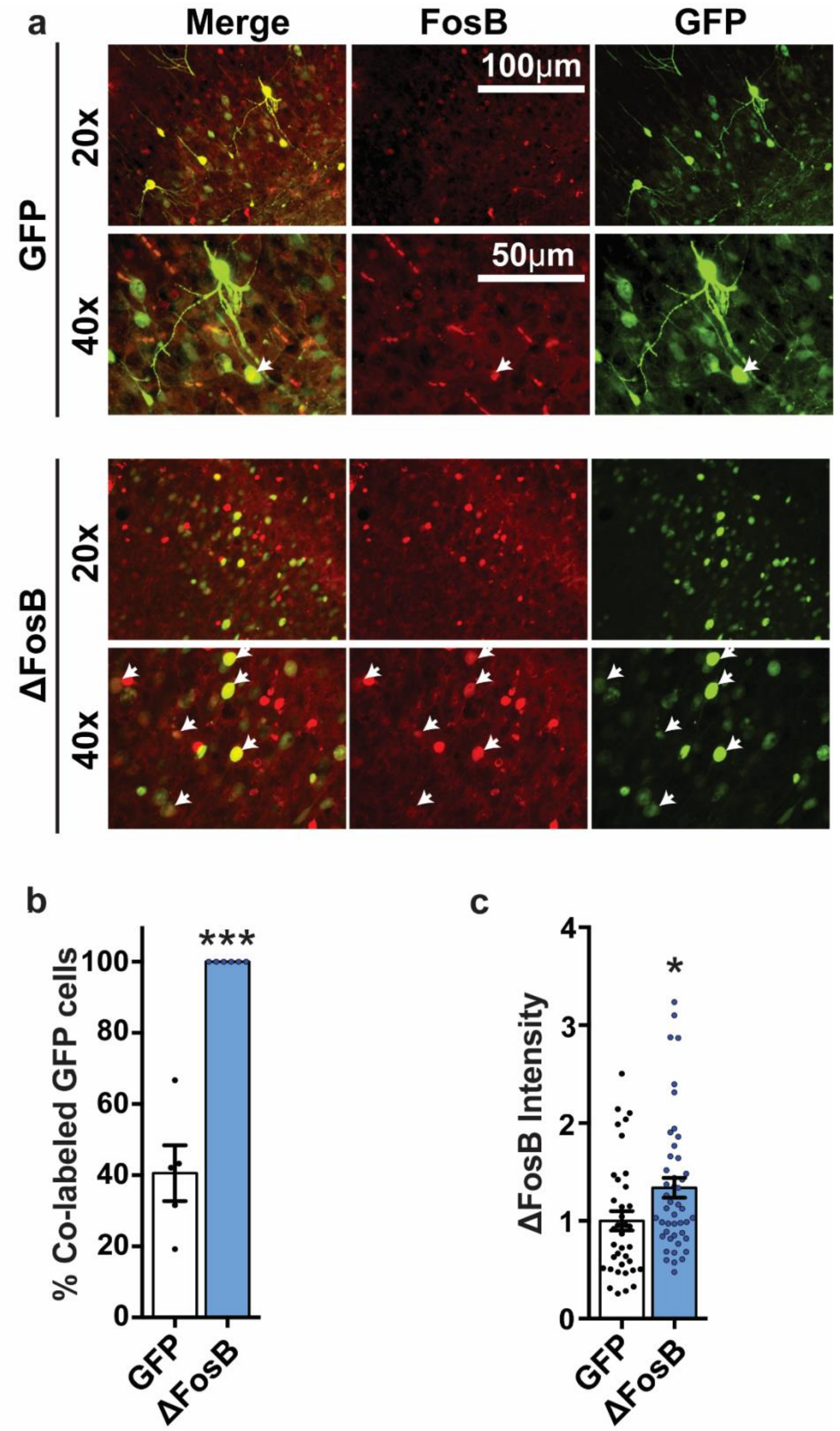
a
Merge
FosB
GFP
100µm
50µm
20x
40x
GFP
ΔFosB
20x
40x
b
% Co-labeled GFP cells
***
0
20
40
60
80
100
GFP
ΔFosB
c
ΔFosB Intensity
*
0
1
2
3
4
GFP
ΔFosB

Fig. S9

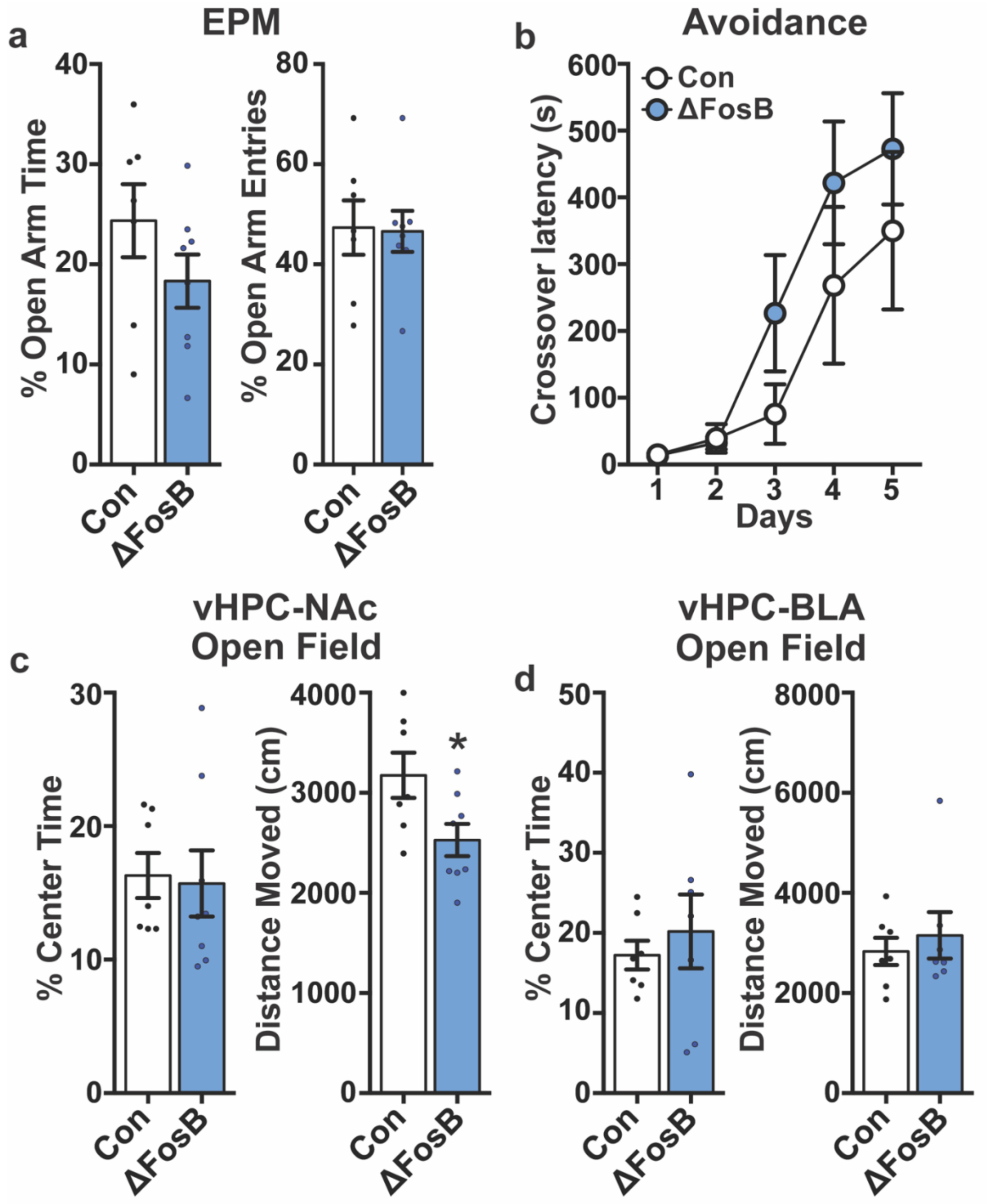

a
EPM
% Open Arm Time
% Open Arm Entries
Con
ΔFosB
b
Avoidance
Crossover latency (s)
Days
c
vHPC-NAc
Open Field
% Center Time
Distance Moved (cm)
d
vHPC-BLA
Open Field

Fig. S10

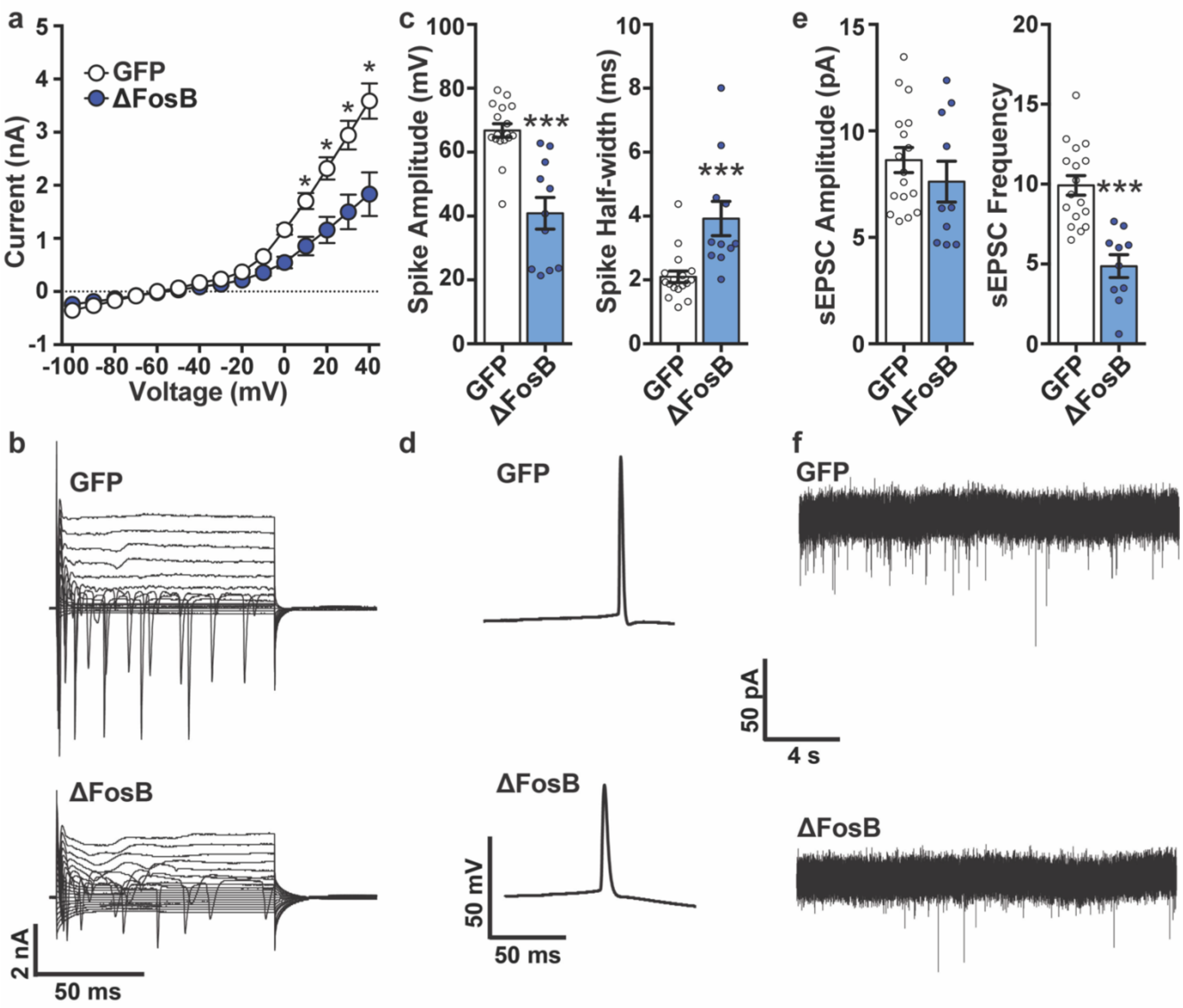
a
GFP
ΔFosB
Current (nA)
Voltage (mV)
b
GFP
ΔFosB
2 nA
50 ms
c
Spike Amplitude (mV)
Spike Half-width (ms)
GFP
ΔFosB
***
d
GFP
ΔFosB
50 mV
50 ms
e
sEPSC Amplitude (pA)
sEPSC Frequency
f
GFP
ΔFosB
50 pA
4 s

Fig. S11

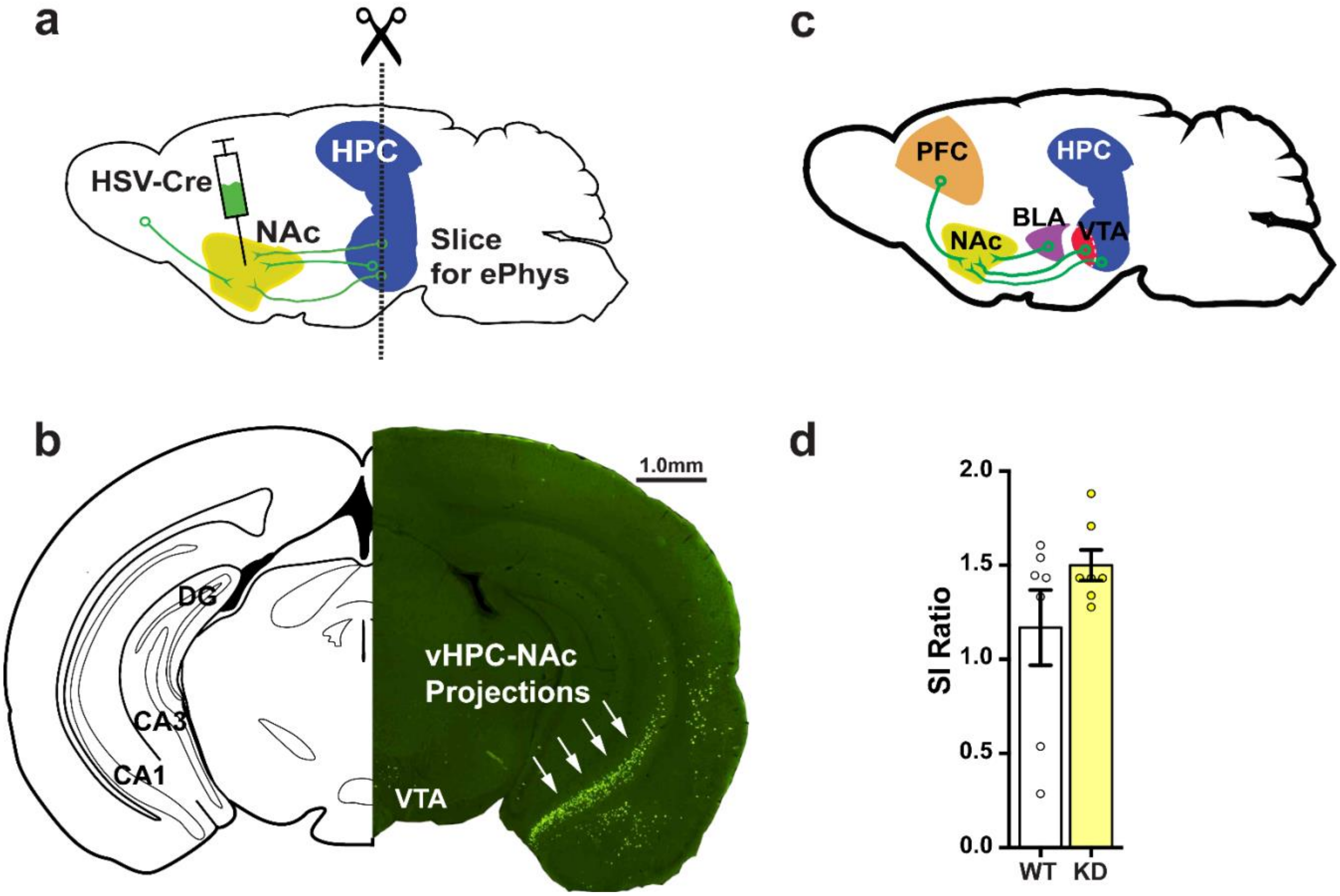
a
HSV-Cre
HPC
NAc
Slice
for ePhys
c
PFC
HPC
BLA
NAc
VTA
b
1.0mm
DG
CA3
CA1
vHPC-NAc
Projections
VTA
d
SI Ratio
2.0
1.5
1.0
0.5
0.0
WT
KD

Fig. S12

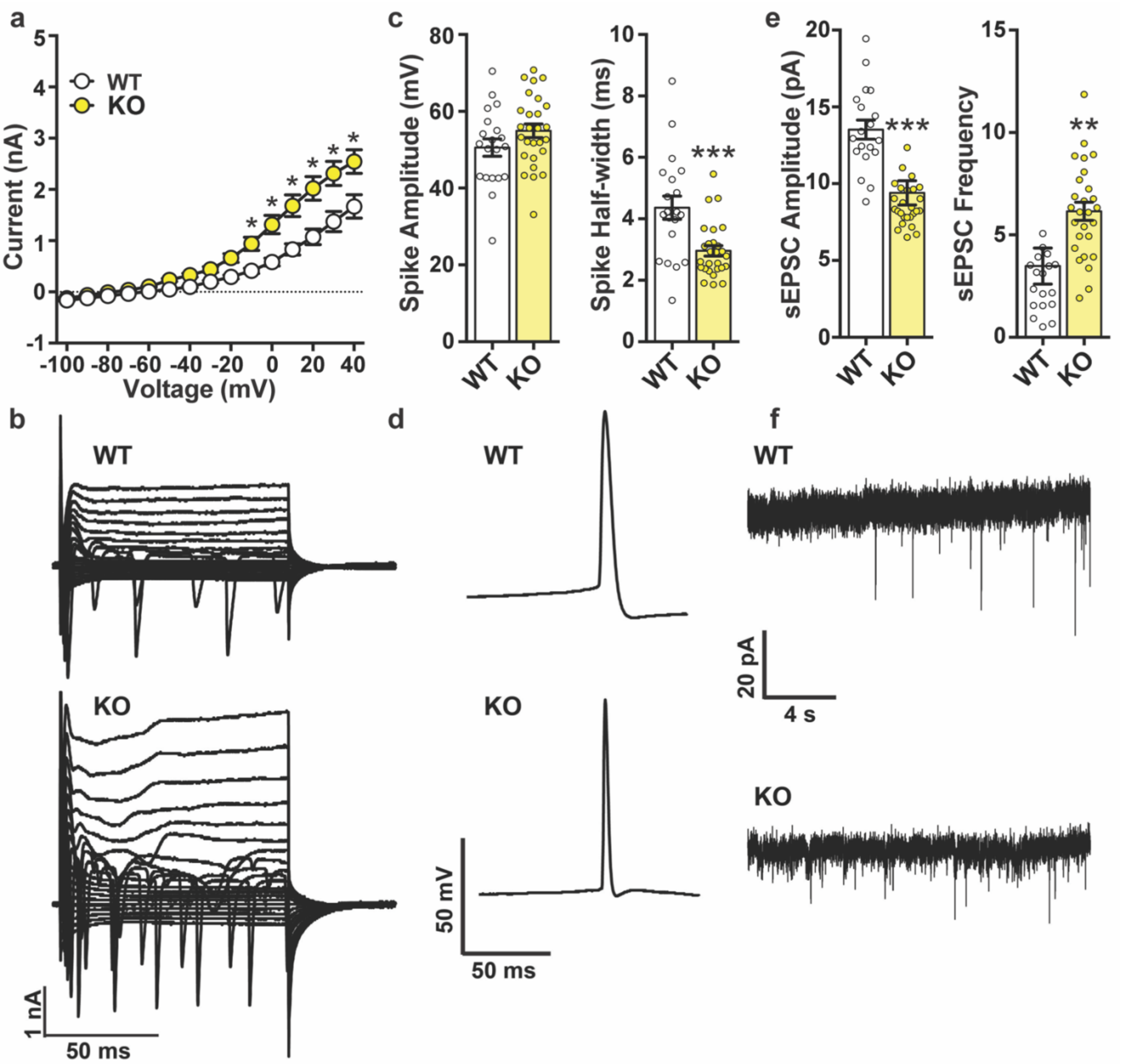

a
WT
KO
Current (nA)
Voltage (mV)
c
Spike Amplitude (mV)
Spike Half-width (ms)
***
e
sEPSC Amplitude (pA)
***
sEPSC Frequency
**
b
WT
KO
1 nA
50 ms
d
WT
KO
50 mV
50 ms
f
WT
20 pA
4 s
KO

Fig. S13

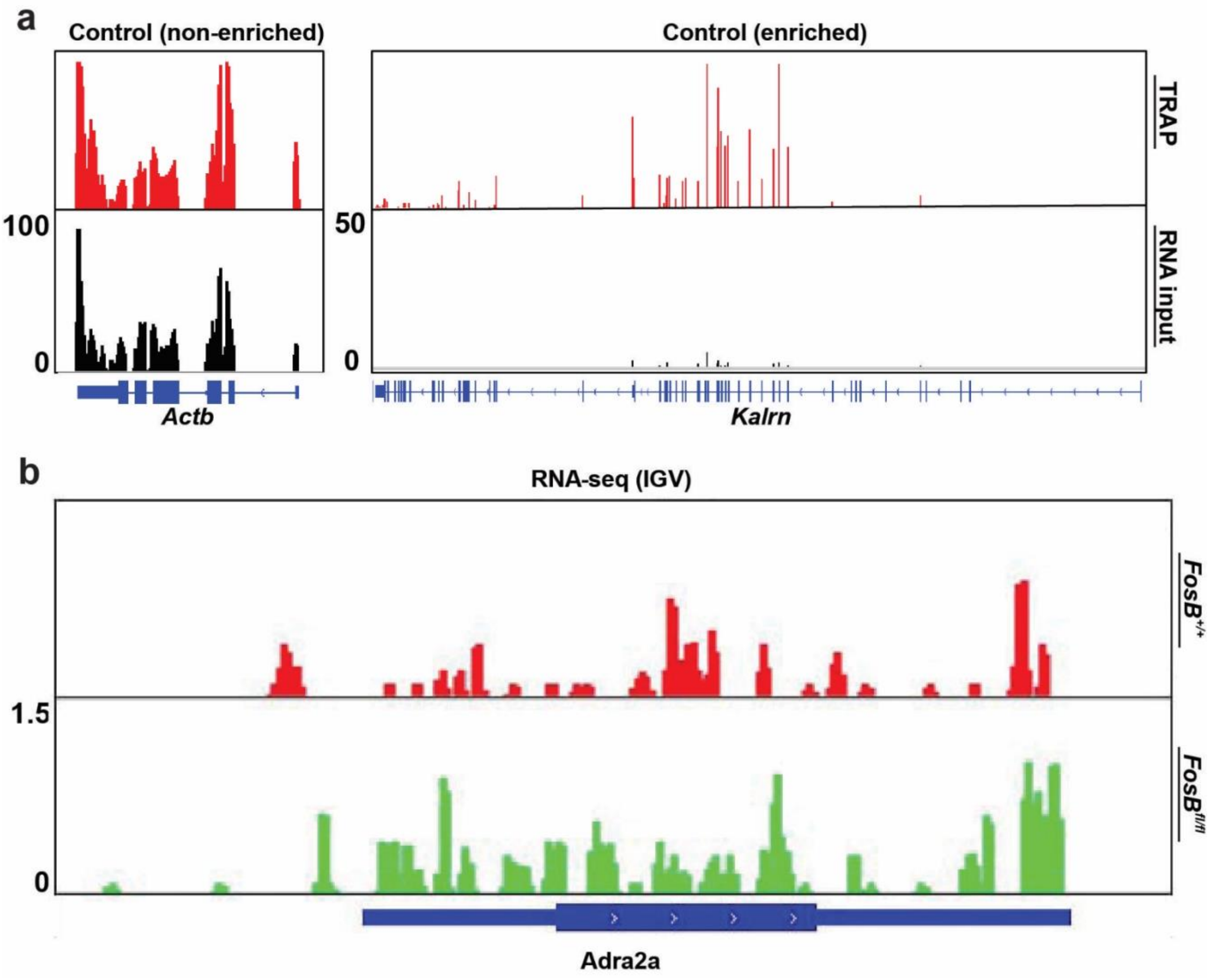
a
Control (non-enriched)
Control (enriched)
TRAP
RNA input
100
0
50
0
Actb
Kalrn
b
RNA-seq (IGV)
FosB+/+
FosBfl/fl
1.5
0
Adra2a
