# Supplementary material for: Circuit-specific hippocampal ΔFosB underlies resilience to stress-induced social avoidance
Source: Nat Commun. 2020 Sep 8;11:4484. doi: 10.1038/s41467-020-17825-x (PMC7479591; doi:10.1038/s41467-020-17825-x)
Supplement: Supplementary file 3 — Reporting Summary [file 41467_2020_17825_MOESM3_ESM.pdf]

nature research

Corresponding author(s): Alfred J. Robison

Last updated by author(s): 20-06-30

Reporting Summary

Nature Research wishes to improve the reproducibility of the work that we publish. This form provides structure for consistency and transparency in reporting. For further information on Nature Research policies, see Authors & Referees and the Editorial Policy Checklist .

Statistics

For all statistical analyses, confirm that the following items are present in the figure legend, table legend, main text, or Methods section.

n/a	Confirmed	
☐	☒	The exact sample size (n) for each experimental group/condition, given as a discrete number and unit of measurement
☐	☒	A statement on whether measurements were taken from distinct samples or whether the same sample was measured repeatedly
☐	☒	The statistical test(s) used AND whether they are one- or two-sided<br>*Only common tests should be described solely by name; describe more complex techniques in the Methods section.*
☒	☐	A description of all covariates tested
☐	☒	A description of any assumptions or corrections, such as tests of normality and adjustment for multiple comparisons
☐	☒	A full description of the statistical parameters including central tendency (e.g. means) or other basic estimates (e.g. regression coefficient) AND variation (e.g. standard deviation) or associated estimates of uncertainty (e.g. confidence intervals)
☐	☒	For null hypothesis testing, the test statistic (e.g. F, t, r) with confidence intervals, effect sizes, degrees of freedom and P value noted<br>*Give P values as exact values whenever suitable.*
☒	☐	For Bayesian analysis, information on the choice of priors and Markov chain Monte Carlo settings
☒	☐	For hierarchical and complex designs, identification of the appropriate level for tests and full reporting of outcomes
☐	☒	Estimates of effect sizes (e.g. Cohen's d, Pearson's r), indicating how they were calculated

Our web collection on statistics for biologists contains articles on many of the points above.

Software and code

Policy information about availability of computer code

Data collection	CleverSys TopScan Realtime 2.0 (Clever Sys Inc, Reston, VA, USA) for automated behavioral tracking<br>Axon pClamp 10 (Molecular Devices, San Jose, CA, USA) for electrophysiological recordings and manipulation
Data analysis	e-CRISP software (open source; http://www.e-crisp.org/E-CRISP/)<br>TopHat 2.1.1 (open source; https://ccb.jhu.edu/software/tophat/index.shtml)<br>HTSseq-counts software 0.12.4 (open source; https://htseq.readthedocs.io/en/master/count.html)<br>DESeq2 3.1.1 (open source; https://bioconductor.org/packages/release/bioc/html/DESeq2.html)<br>CleverSys TopScan 2.0 (Clever Sys Inc, Reston, VA, USA) for behavioral analysis<br>Clampfit 10 (Molecular Devices, San Jose, CA, USA) for electrophysiological data analysis<br>Mini Analysis 6 (Synaptosoft, Inc, Fort Lee, NJ, USA) for anaylsis of spontaneous recordings<br>ImageJ (1.52d, NIH) for analysis of histological immunostaining and Western blots<br>Prism 6 & 7 (Graphpad, San Diego, CA) for all statistical analysis and graphing

For manuscripts utilizing custom algorithms or software that are central to the research but not yet described in published literature, software must be made available to editors/reviewers. We strongly encourage code deposition in a community repository (e.g. GitHub). See the Nature Research guidelines for submitting code & software for further information.

Data

Policy information about availability of data

All manuscripts must include a data availability statement. This statement should provide the following information, where applicable:

- Accession codes, unique identifiers, or web links for publicly available datasets
- A list of figures that have associated raw data
- A description of any restrictions on data availability

Sequencing datasets generated during and analyzed during the current study are available in the NIH GEO repository (https://www.ncbi.nlm.nih.gov/geo/) with the

accession code GSE137283. All the other data supporting the findings of this study are available within the article and its supplementary information files and from the corresponding author upon reasonable request. A reporting summary for this article is available as a Supplementary Information file.

Field-specific reporting

Please select the one below that is the best fit for your research. If you are not sure, read the appropriate sections before making your selection.

- [x] Life sciences
- [ ] Behavioural & social sciences
- [ ] Ecological, evolutionary & environmental sciences

For a reference copy of the document with all sections, see nature.com/documents/nr-reporting-summary-flat.pdf

Life sciences study design

All studies must disclose on these points even when the disclosure is negative.

Sample size

Adequate sample sizes are generally determined based upon inter-sample variability. Throughout the manuscript, we determined the significance of results based upon a general confidence interval of 95%. We do not include specific justifications of sample size within the methods (e.g., power analyses), as sample sizes were based on extensive laboratory experience with these endpoints.

Data exclusions

Data from electrophysiological recordings were excluded based on specific cellular properties (e.g. resting membrane potential, series/access resistance, input resistance) that indicated unhealthy cells or an unstable patch. These were pre-established criteria based on cellular properties to exclude data prior to analysis. One data point from Figure 5c (Kctd9) was excluded prior to analysis due to plate contamination. Plate contamination was a pre-established criteria to exclude data prior to analysis. No other data were excluded from the analyses.

Replication

Behavioral experiments were often replicated in two to three cohorts (conducted at separate times). All replications were successful and showed the same pattern as described in the published figures. In addition, all immunostaining was replicated at least twice. Electrophysiology results represent at least 4 biological replicates (i.e., mice) for each experiment, though the unit of statistical analysis is cells, as the viral gene manipulation being performed is a manipulation of the individual cells, not of the entire animal. TRAP RNA sequencing experiments were performed only once, though there were 3-4 mice pooled for each of three samples per group thus ensuring the inclusion of many biological replicates. Moreover, RNAseq results that are interpreted in this manuscript (i.e., Adra2a and others) are validated using both cell culture (Fig 5c) and viral manipulation (Fig 5e&f) in mouse brain.

Randomization

All mice were randomized into groups.

Blinding

Blinding was used for all immunohistochemistry and electrophysiological analyses and most behavioral experiments. Some behavior experiments lacked blinding as the same experimenter performed both the surgical manipulation and the behavioral experiments. However, when this occurred, experiments were performed in multiple cohorts each by a different experimenter, to ensure the robustness and reliability of the results. Moreover, behaviors were not scored subjectively, but rather by digital video tracking software (Cleversys), thus removing any potential experimenter bias, regardless of blinding.

Reporting for specific materials, systems and methods

We require information from authors about some types of materials, experimental systems and methods used in many studies. Here, indicate whether each material, system or method listed is relevant to your study. If you are not sure if a list item applies to your research, read the appropriate section before selecting a response.

Materials & experimental systems

n/a	Involved in the study	
[ ]	[x]	Antibodies
[ ]	[x]	Eukaryotic cell lines
[x]	[ ]	Palaeontology
[ ]	[x]	Animals and other organisms
[x]	[ ]	Human research participants
[x]	[ ]	Clinical data

Methods

n/a	Involved in the study	
[x]	[ ]	ChIP-seq
[x]	[ ]	Flow cytometry
[x]	[ ]	MRI-based neuroimaging

Antibodies

Antibodies used

anti-FosB 5G4 Rabbit mAb (2251; Cell Signaling Technology)
anti-FosB [83B1138] Mouse mAb (ab11959, Abcam)
anti-GFP Goat pAb (ab5450; Abcam)
anti-alpha 2A adrenergic receptor Rabbit pAb (PA1-048; Invitrogen)
anti-GFP mouse mAbs (Memorial Sloan-Kettering Monoclonal Antibody Facility; clone names: Htz-GFP-19F7 and Htz-GFP-19C8)
anti-Cas9 mouse mAb (C15200229; Diagenode, lot 001)
Goat Anti-Rabbit IgG Antibody (H+L) Peroxidase (PI-1000; Vector)
Goat Anti-Rabbit IgG Antibody (H+L) Biotinylated (BA-1000; Vector)

Alexa Fluor® 488 AffiniPure Donkey Anti-Goat IgG (H+L) (pAb; 705-545-147; Jackson)
Cy™3 AffiniPure Donkey Anti-Mouse IgG (H+L) (pAb; 715-165-150; Jackson)
Cy™5 AffiniPure Goat Anti-Mouse IgG (H+L) (pAb; 115-175-146; Jackson)
Cy™3 AffiniPure Donkey Anti-Rabbit IgG (H+L) (pAb; 711-165-152; Jackson)
Cy™5 AffiniPure Donkey Anti-Rabbit IgG (H+L) (pAb; 711-175-152; Jackson)

Validation

Validation of commercial antibodies are found on the suppliers' webpages. Validation of Memorial Sloan-Kettering antibodies can be found at The Antibody Registry (antibodyregistry.org) under AB_2716736 and AB_2716737. Validation in lab was conducted using no primary controls in all immunohistochemistry experiments. Validation of antibody concentration was conducted in lab and either followed by manufacturer recommendation or validated in preliminary tests in sample tissue across varying concentrations.

Anti-FosB 5G4 (Cell Signaling 2251) has been extensively validated, including in Western blotting and immunostaining (as conducted in the current publication). Knockout mice generated by Yutsudo et al., (2013; Neurospsychopharmacology) were used to ensure that FosB-null mice did not display bands representing FosB gene products (FosB at 43 kD, ΔFosB at 37 kD, and Δ2ΔFosB at 24 kD) using anti-FosB 5G4 in Western blot. We have also shown that the signal generated by this antibody is not present in floxed FosB dentate gyrus neurons expressing Cre recombinase (Manning et al., 2018, Neuroscience). In addition, it has been validated for immunohistochemistry/immunofluorescence in the following articles:
• Ozek, C., Krolewski, R.C., Buchanan, S.M. et al. Growth Differentiation Factor 11 treatment leads to neuronal and vascular improvements in the hippocampus of aged mice. Sci Rep 8, 17293 (2018). https://doi.org/10.1038/s41598-018-35716-6
• Aran, D., Camarda, R., Odegaard, J. et al. Comprehensive analysis of normal adjacent to tumor transcriptomes. Nat Commun 8, 1077 (2017). https://doi.org/10.1038/s41467-017-01027-z

Anti-FosB [83B1138] (Abcam ab11959) has been extensively validated, including in Western blotting and immunostaining (as conducted in the current publication). We have used this antibody to show that viral overexpression of ΔFosB in dorsal hippocampal CA1 neurons increases FosB/ΔFosB expression observed by immunofluorescent staining (Eagle et al., 2015, J Neurosci). Abcam also cites 22 references that have used this antibody for Western blotting and immunohistochemistry (as is used in the current paper). Of particular note, we have used a Cre-dependent engineered zinc finger protein targeted to the FosB promoter and fused to the transcriptional repressor G9a that was injected into nucleus accumbens and found that FosB expression (stained using this antibody) was reduced in only Cre-expressing nucleus accumbens neurons (Hamilton et al., 2017, Neuropsychopharmacology).

Anti-GFP (Abcam ab5450) has been extensively validated in Western blotting, immunocytochemistry, immunohistochemistry (whole mount, fixed frozen, formalin/pfa-xied paraffin-embedded), immunocytochemistry, immunofluorescence, and electron microscopy. We have published 2 articles showing that it enhances native GFP signal in hippocampus (Manning et al., 2019, Neuroscience; Williams et al., 2020, Biol Psychiatry). Supplier website (https://www.abcam.com/gfp-antibody-ab5450.html) also shows ab5450 staining GFP in GFP-transfected NIH3T3 cells. The supplier includes 11 reviews and 136 publications referencing this antibody, including:
• Corbett BF et al. Sphingosine-1-phosphate receptor 3 in the medial prefrontal cortex promotes stress resilience by reducing inflammatory processes. Nat Commun 10:3146 (2019).
• Redd MA et al. Patterned human microvascular grafts enable rapid vascularization and increase perfusion in infarcted rat hearts. Nat Commun 10:584 (2019).
• Hamilton PJ et al. Cell-Type-Specific Epigenetic Editing at the Fosb Gene Controls Susceptibility to Social Defeat Stress. Neuropsychopharmacology 43:272-284 (2018).

Anti-alpha 2A adrenergic receptor antibody (Invitrogen, PA1-048) has been well validated in Western blotting and immunohistochemistry. The supplier reports Adra2a expression by Western blotting using this antibody in PC-3, HEK-293, T47D, and HEK 92.1.7 cells at 60 and 50 kDa. The supplier also provides 8 references citing this antibody in use including showing expression of Adra2a in muscle, brain, and cochlea. In addition, immunostaining using this antibody has been published to identify Adra2a neurons in human cortical plate (Pollen et al., 2014, Nat Biotech), western blotting of Adra2a in rat ileum and colon (Blandizzi et al., 2003, Br J Pharmacol).

We validated the anti-GFP (Memorial Sloan-Kettering Monoclonal Antibody Facility, clone names: Htz-GFP-19F7 and Htz-GFP-19C8) in TRAP by performing immunoprecipitation using this antibody in non-L10-GFP-expressing tissue, and showing no detectable RNA precipitation. These antibodies have been used extensively, including publication in high impact journals, for TRAP experiments. These include:
• Heiman M, Kulicke R, Fenster RJ, Greengard P, Heintz N. Cell type-specific mRNA purification by translating ribosome affinity purification (TRAP). Nat Protoc. 2014;9(6):1282-1291. doi:10.1038/nprot.2014.085
• Burger LL, Vanacker C, Phumsatitpong C, et al. Identification of Genes Enriched in GnRH Neurons by Translating Ribosome Affinity Purification and RNAseq in Mice. Endocrinology. 2018;159(4):1922-1940. doi:10.1210/en.2018-00001
• Zhang Z, Zhong P, Hu F, et al. An Excitatory Circuit in the Perioculomotor Midbrain for Non-REM Sleep Control. Cell. 2019;177(5):1293-1307.e16. doi:10.1016/j.cell.2019.03.041
• Michalovicz LT, Kelly KA, Vashishtha S, et al. Astrocyte-specific transcriptome analysis using the ALDH1L1 bacTRAP mouse reveals novel biomarkers of astrogliosis in response to neurotoxicity. J Neurochem. 2019;150(4):420-440. doi:10.1111/jnc.14800
• Ostroff LE, Santini E, Sears R, et al. Axon TRAP reveals learning-associated alterations in cortical axonal mRNAs in the lateral amgydala. Elife. 2019;8:e51607. Published 2019 Dec 11. doi:10.7554/eLife.51607
• Sawicka K, Hale CR, Park CY, et al. FMRP has a cell-type-specific role in CA1 pyramidal neurons to regulate autism-related transcripts and circadian memory. Elife. 2019;8:e46919. Published 2019 Dec 20. doi:10.7554/eLife.46919
• Groschner LN, Chan Wah Hak L, Bogacz R, DasGupta S, Miesenböck G. Dendritic Integration of Sensory Evidence in Perceptual

Decision-Making. Cell. 2018;173(4):894-905.e13. doi:10.1016/j.cell.2018.03.075

Validation of Memorial Sloan-Kettering antibodies can be found at The Antibody Registry (antibodyregistry.org) under AB_2716736 and AB_2716737. Validation in lab was conducted using no primary controls in all immunohistochemistry experiments. Validation of antibody concentration was conducted in lab and either followed by manufacturer recommendation or validated in preliminary tests in sample tissue across varying concentrations.

Anti-Cas9 (C15200229, Diagenode) has been well-validated in ChIP, Western blotting, IP, and immunostaining. ChIP was performed on NIH3T3 cells stably expressing GFP-H2B, nuclease dead Cas9, and a GFP-targeting gRNA. Western blot was performed on protein extracts from HeLa cells transfected with Cas9. The supplier also reports 5 publications that have used this antibody including:

• Byrne, S.M. and Church, G.M. 2015. CRISPR-mediated gene targeting of human induced pluripotent stem cells. Curr. Protoc. Stem Cell Biol. 35: 5A.8.1- 5A.8.22. doi: 10.1002/9780470151808.sc05a08s35

• Hu, Y. et al. RNA-Based dCas9–VP64 System Improves the Viability of Cryopreserved Mammalian Cells. Nano LIFE 08, 1850004, doi:10.1142/S1793984418500046 (2018).

Eukaryotic cell lines

Policy information about cell lines

Cell line source(s)	Neuro2A (ATCC CCL31, lot 61793985)
Authentication	Neuro2A cells were purchased directly from ATCC, stock number CCL-131, lot 61793985. Initial passage is number 181, and cells were passed no more than 10 additional times before discarding and plating a fresh stock from the manufacturer. Karyotype is confirmed by the vendor, and cell type is re-confirmed in each passage by morphology and adherence.
Mycoplasma contamination	None was detected. Test for contamination was conducted by the supplier and can be found in the Certificate of Analysis for the lot number 61793985 on the supplier's website (https://www.atcc.org/~/media/Files/Certificates%20of%20Analysis/1/A/8/8/CCL-131_61793985.ashx).
Commonly misidentified lines (See ICLAC register)	No commonly misidentified lines were used in this study.

Animals and other organisms

Policy information about studies involving animals; ARRIVE guidelines recommended for reporting animal research

Laboratory animals	C57Bl6/J male mice were 7-8 wks upon arrival from Jackson Labs. The floxed FosB mouse strain (FosBfl/fl) is a generous gift from the laboratory of Dr. Eric Nestler at the Icahn School of Medicine at Mount Sinai and come from a mixed C57Bl/6J background. Both males and female F1 breeders were received from Dr. Eric Nestler. The Rosa26eGFP-L10a male and female F1 breeder mice were a generous gift from the laboratory of Dr. Gina Leinninger at Michigan State University and were backcrossed to a C57Bl/6J background. CD-1 retired male breeder mice (age varies) from Charles River were also used for chronic social defeat stress experiments.
Wild animals	No wild animals were used in this study.
Field-collected samples	No field-collected samples were used in this study.
Ethics oversight	All experiments were approved by the Institutional Animal Care and Use Committee at Michigan State University in accordance with AAALAC

Note that full information on the approval of the study protocol must also be provided in the manuscript.
